# Strengthening Reinforced Concrete Members Using FRP—Evaluating Fire Performance, Challenges, and Future Research Directions: A State-of-the-Art Review

**DOI:** 10.3390/polym17010013

**Published:** 2024-12-25

**Authors:** Mahmood Haris, Ergang Xiong, Wanyang Gao, Mabor Achol Samuel, Najam Us Sahar, Anwar Saleem

**Affiliations:** 1 School of Civil Engineering, Chang’an University, Xi’an 710061, China; harismahmood@chd.edu.cn (M.H.); maborachol@chd.edu.cn (M.A.S.); wellwellwell111@yahoo.com (N.U.S.); anwarsaleem@chd.edu.cn (A.S.); 2 School of Naval Architecture, Ocean and Civil Engineering, Shanghai Jiao Tong University, Shanghai 200240, China; wanyanggao@sjtu.edu.cn

**Keywords:** fiber-reinforced polymers (FRPs) composites, RC members, strengthening, fire resistance, finite element modeling, elevated temperature

## Abstract

Fiber-reinforced polymer (FRP) composites are increasingly used in civil engineering for strengthening and repairing existing reinforced concrete (RC) members using externally bonded reinforcement (EBR) and near-surface mounted (NSM) methods. However, the fire performance of FRP-strengthened RC members has been an important issue that should be properly considered in the fire safety design process since FRP composites exhibit significant performance degradation at elevated temperatures. This paper aims to review studies on the fire performance of FRP-strengthened RC members based on the existing research results presented in the literature to provide a comprehensive understanding of key factors influencing the structural behavior of FRP-strengthened RC members under fire conditions. It provides an overview of FRP composite material properties, such as their mechanical and thermal behavior and bond characteristics between FRP-to-concrete interfaces at elevated temperatures. Additionally, this paper reviews experimental and numerical research conducted on FRP-strengthened RC members, examining load-carrying capacities and fire endurance ratings. Finally, this review will provide existing fire resistance design methods as well as simple design methods for temperature prediction.

## 1. Introduction

During the 1960s and 1970s, fiber-reinforced polymer (FRP) composites were primarily introduced in marine, aerospace, and automotive industries after finding that they could be applied to a variety of applications. FRP composites have gained popularity among civil engineers since the mid-1990s due to the fact that FRP composites provide significant strength improvements compared to conventional steel reinforcements due to their outstanding strength and stiffness. Today’s world keeps constantly changing, and more effective and affordable materials are being sought for engineering purposes. Recently, textile-reinforced mortar (TRM) has emerged as an alternative to FRP composites for structures exposed to high temperatures. Depending on the requirements of the application, both FRP and TRM systems offer distinct advantages in structural strengthening. Originally, FRP composites were used to strengthen outdoor structures, such as naturally ventilated parking garages and bridges, where fire safety has little concern as they were naturally ventilated [1]. However, in the case of indoor FRP-strengthened RC members, fire safety becomes an issue in order to meet fire performance requirements as specified in building codes and standards since FRP composites have weaknesses, such as poor fire performance (rapid deterioration in bond and mechanical properties, flammability, and smoke generation under fire conditions).

Consequently, it becomes necessary to investigate the fire performance of FRP composites and FRP-strengthened RC members in RC buildings under fire conditions. Besides, TRM has become increasingly popular in high-temperature environments due to its cement- or lime-based mortar matrix, which provides superior resistance to heat and fire compared to epoxy resin matrices in FRP composites. TRM’s thermal stability, compatibility with concrete and masonry, and adaptability to complex geometries make it ideal for structural confinement in fire-prone areas. Although TRM performs well in thermal environments, FRP’s superior strength, chemical resistance, and lightweight properties make it the preferred material for structural rehabilitation and strengthening. To enhance the fire performance of FRP-strengthened RC members at elevated temperatures, they should often be ensured using fire protection systems.

Fire protection systems for FRP-strengthened RC members play a critical role in protecting the materials and structural integrity of buildings and infrastructure during fire exposure. Fire protection systems generally include active and passive protection. Passive fire protection aims to prevent or postpone fire damage using materials (i.e., insulating layers, cementitious encapsulations, and fire-resistant coatings). Passive fire protection provides a thermal barrier, preventing excessive temperature elevation in FRP composite to ensure its mechanical properties at elevated temperatures. In contrast, active fire protection includes real-time surveillance and immediate response mechanisms such as fire alarms, sprinkler systems, and extinguishing technologies that identify and mitigate fire before major structural damage occurs. Integrating both passive and active fire protection systems significantly enhances the fire safety of FRP-strengthened RC members. As an example, combining both cementitious fireproofing and sprinkler methods provides both long-term passive protection and rapid-fire protection in buildings and infrastructure [2]. A combination of intumescent coatings and fire detection systems provides a balanced solution for both passive protection and rapid-fire protection in more confined or industrial areas [3]. Integrating both passive and active systems, commonly known as hybrid fire protection systems, provides optimal fire protection for buildings subjected to fire conditions, improving both fire resistance and reducing fire-related failure risks [4]. Overall, integrating both passive and active fire protection systems ensures the performance, safety, and durability of buildings and infrastructure, thus offering a superior solution for fire protection that prevents material failure risk and maintains structural integrity under fire conditions.

FRP composites are usually incorporated to strengthen RC members using the externally bonded reinforcement (EBR) method, where prefabricated FRP components (i.e., sheets or laminates) are wrapped around RC members. Alternatively, the near-surface mounted (NSM) method can also be used to strengthen RC members, where steel rebars are mounted near the surface of the RC members. Additionally, another way of enhancing the structural stability of RC members can be achieved by implementing FRP rebars as internal reinforcement. During the pultrusion process of these FRP rebars, fibers are pulled through resin baths and heated through dies to produce strong, lightweight, corrosion-resistant FRP rebars. These FRP rebars provide eight times the strength in comparison to conventional steel reinforcement bars. Further, as FRP composites are exposed to moderately high temperatures, the resin of FRP composites transforms from a hard and brittle state to a more viscous or rubbery state; this is called the glass transition process, also known as glass transition temperature ($T_{g}$). Without a doubt, ($T_{g}$) plays a key factor that significantly affects FRP’s thermal, mechanical, and bonding properties and must not be overlooked.

This paper provides an up-to-date, State-of-the-Art review regarding the fire behavior of FRP materials and their use in strengthening RC structural elements using FRP composites. The review is based on several key factors that are central to its focus. This review provides summaries of existing research regarding the mechanical properties (i.e., elastic modulus and tensile strength properties as well as ($T_{g}$) influence) and thermal properties of FRP composites at elevated temperatures. As part of this review, we highlight the potential performance of near-surface mounted (NSM) FRP techniques in comparison with externally bonded reinforcement (EBR) FRP techniques, with particular concern regarding bond behavior at FRP-to-Concrete Interface under elevated temperature. Additionally, a comprehensive review of existing fire performance tests and numerical modeling on FRP-strengthened RC members (e.g., columns, beams, and slabs) at elevated temperatures has been reviewed. Finally, this review provides a simple design-oriented approach for the development of a reliable FE strategy that can help predict temperature distribution within bare and insulated FRP-strengthened RC elements under fire conditions, as well as recommendations for future research.

## 2. Fiber Reinforced Polymer (FRP) Materials Properties at Elevated Temperature

### 2.1. Summary of Existing Mechanical Properties

Over the past two decades, several researchers have studied the influence of FRP composite’s mechanical properties at elevated temperatures that are unknown, such as elastic modulus and tensile strength properties as well as glass transition temperature ($T_{g}$) [5,6,7]. Although there are many publications relevant to this topic that have been completed in recent years are also available (e.g., refs. [2,3,8,9,10,11,12,13,14,15,16,17,18,19,20,21,22,23,24,25,26,27,28,29,30,31,32,33]). During the manufacturing of FRP-strengthened RC members, different types and shapes of FRP composites can be used effectively. Many recent studies have focused on differentiating between the ultimate tensile strength and elasticity modulus properties of FRP composites at various temperatures. However, for a comprehensive understanding of the mechanical properties of FRP under fire conditions, ${(T}_{g}$) it must be specified. In FRP composites, there is no doubt that ($T_{g}$) is a key factor influencing polymer matrix performance, particularly when subjected to elevated temperatures. As FRP composites are exposed to moderately high temperatures, the resin of FRP composites transforms from a hard and brittle state to a more viscous or rubbery state; this is called the glass transition process. There is a significant impact on the thermal, mechanical, and bonding properties of FRP as a result of the glass transition. Therefore, it is important to understand glass transition temperature ($T_{g}$) of polymer matrixes to predict its mechanical behavior at elevated temperatures. Typical ($T_{g}$) range for FRP bars, FRP plates, and FRP sheets are 60 °C to 150 °C, 70 °C to 200 °C, and 50 °C to 130 °C, respectively [34,35]. Furthermore, glass transition temperatures ($T_{g}$) can vary based on factors such as polymer type, curing conditions, additives, and the presence of additives/fillers. To ensure that the polymer matrix maintains its mechanical properties within the desired operating range, engineers and material scientists must carefully consider these factors when designing FRP composites. Experimental methods such as differential scanning calorimetry (DSC) and dynamic mechanical analysis (DMA) are the most commonly used methods to measure $(T_{g}$). FRP elongation at break varies significantly depending on the fiber type, matrix material, and shape (i.e., bars, plates, or sheets). CFRP generally exhibits the lowest elongation at break, ranging between 0.5% and 1.5%. On the other hand, GFRP exhibits significantly higher elongation at break, ranging from 1.5% to 3%. With values typically ranging from 1.5% to 2.5%, BFRP composites fall between CFRP and GFRP in terms of elongation at break. Table 1 summarizes the collected data regarding elasticity modulus and tensile strength for the specific targeted area of FRP mechanical properties at elevated temperatures. During these studies, values of maximum temperature $\left(T_{max}\right)$, minimum temperature ${(T}_{min}$), increment in temperature exposure ${(T}_{inc}$), and glass transition temperature $\left(T_{g}\right)$ of FRP mechanical properties have also been provided when available.

**Figure 1:**
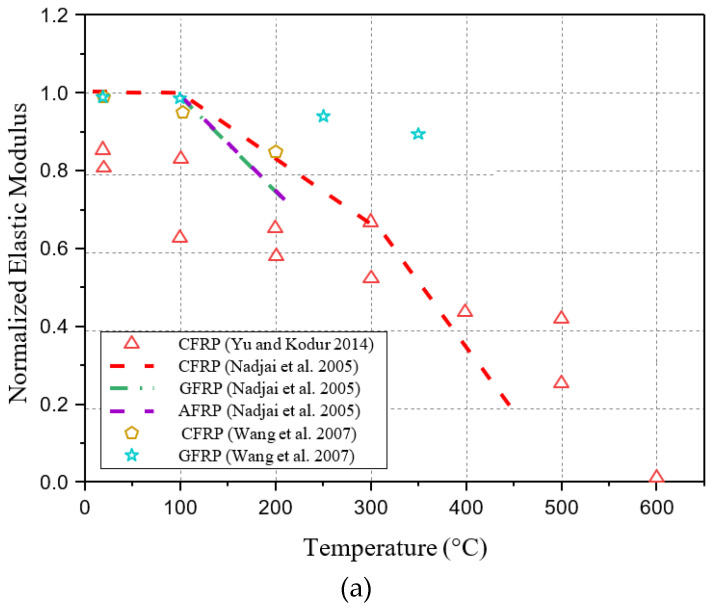

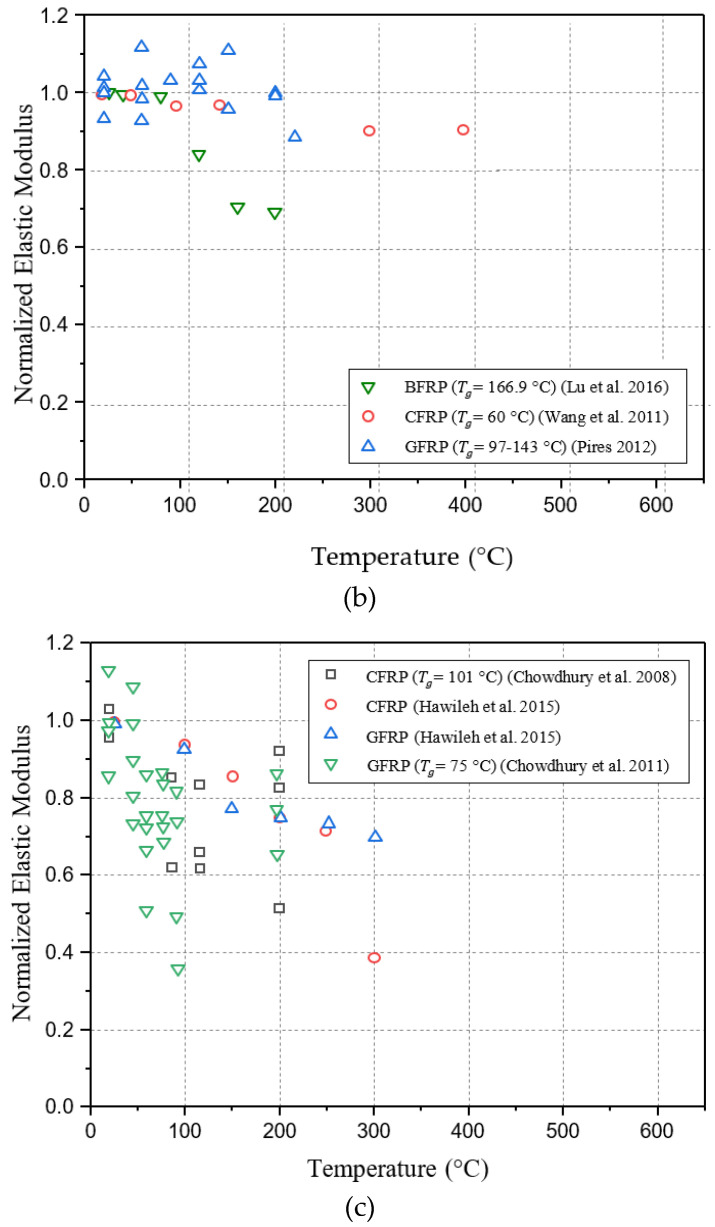
Normalized elastic modulus of FRP materials (i.e., bars, plates, sheets) at elevated temperatures. (**a**) Bars; (**b**) Plates; (**c**) Sheets (Refs. [9,11,12,18,27,28,30,31,33]).

**Figure 2:**
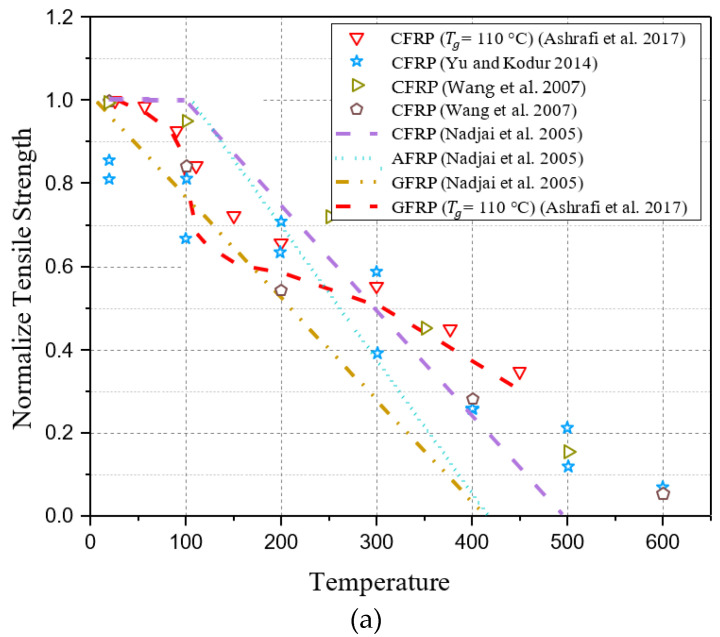

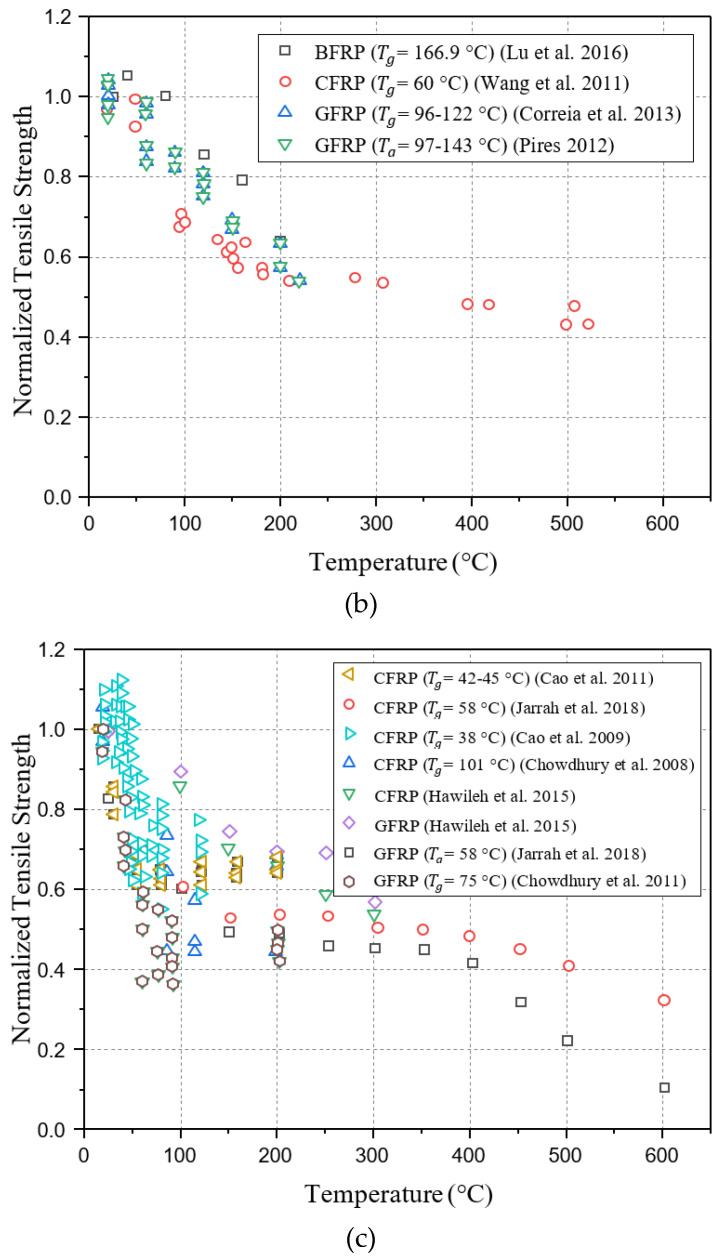
Normalized tensile strengths of FRP materials (i.e., bars, plates, sheets) at elevated temperatures. (**a**) Bars; (**b**) Plates; (**c**) Sheets (Refs. [2,8,9,10,11,12,18,27,28,29,30,31,32,33]).

Figure 1 summarizes elastic modulus data from tensile coupons tested on different types of FRP bars at elevated temperatures [18,27,28]. Moreover, it includes the results of the tests conducted by [30,31] on FRP plates and [9,11,12] on FRP sheets for comparison. In these studies, the FRP composite’s elastic modulus values were normalized by comparing them to those obtained at different temperatures. The results indicate that FRP sheets degrade more severely than FRP bars or plates in terms of elastic modulus. Due to the fact that the performance of FRP laminates depends primarily on the glass transition temperature of the polymer matrix ($T_{g/p}$), this temperature needs to be considered when designing mathematical models of elastic modulus degradation at elevated temperatures. Figure 2 summarizes the available data on FRP bars tensile strength for different types of FRP bars conducted by [18,27,28,29] at elevated temperatures. In a similar way to Figure 1, it also includes test results for FRP plates conducted by [30,31,32,33] and FRP sheets conducted by [2,8,9,10,11,12] for comparison. The tensile modulus of composite FRP was normalized by comparing it to the values obtained at various temperatures during these studies. Based on these figures, FRP sheets degrade more significantly in tensile strength than FRP plates or bars. Similarly, it may be due to the fact of glass transition temperature of the polymer matrix ($T_{g/p}$).

It should be noted that the results listed above are relevant to the fiber type selected, fiber volume fraction, and manufacturing conditions of the tested FRP, making direct comparisons challenging. As a result of reviewing the results, several observations were made: Temperature affects FRP tensile strength and modulus significantly, initially declining sharply as a result of resin matrix glass transition, then gradually decreasing with matrix decomposition, and finally, a sharp drop with fiber oxidation that begins at around 500 °C and intensifies between 600 and 800 °C; despite the resin matrix undergoing glass transition and decomposition, the fibers retain a substantial portion of their tensile properties; results from steady-state and transient tests are comparable; further experimental investigation may be needed to determine how FRP sheets lose tensile strength and elastic modulus at elevated temperatures.

**Table 1:** Mechanical properties of FRP materials at elevated temperatures.

References	Material	FRP Shape	Glass Transition		Tensile Strength			Elastic Modulus	
			$T_{g}$ [°C]	Methods	$T_{min}$	$T_{max}$	$T_{inc}$	$T_{min}$	$T_{max}$
Wong et al. (2004) [13]	E-glass FRP	Plates	—	—	20	250	—	20	250 ^1^
Nadjai et al. (2005) [28]	CFRP	Bars	—	—	20	475	—	20	500 ^2^
	GFRP	Bars	—	—	0	400	—	20	400 ^2^
	AFRP	Bars	—	—	20	400	—	20	500 ^2^
Wang et al. (2007) [27]	CFRP	Bars	—	—	20	600	100	20	200
	GFRP	Bars	—	—	20	500	150	20	350
Chowdhury et al. (2008) [9]	CFRP	Sheets	101	DSC	20	200	—	20	200
Bai (2009), Bai et al. (2007), Bai et al. (2008), Bai and Keller (2009) [15,16,17,19]	GFRP	Plates	110	DMA	20	220	—	20	220
Cao and Wu (2008) [14]	CFRP	Plates	38	DMA	16	200	—	16	200
	GFRP								
	BFRP								
Cao et al. (2009) [8]	CFRP	Sheets	38	DMA	16	200	—	—	—
Cao et al. (2011) [2]	CFRP	Sheets	42–45	DMA	20	120	—	—	—
Wang et al. (2011) [31]	CFRP	Strips	—	—	20	700	—	20	700 ^1^
	CFRP	Plates	60	—	20	700	—	20	700 ^1^
Chowdhury et al. (2011) [11]	GFRP	Sheets	75	DSC	20	200	—	20	200
Pires (2012) [33]	GFRP	Plates	97–143	DMA /DSC	20	250	—	20	250 ^1^
Correia et al. (2013) [32]	GFRP	Plates	96–122	DMA	20	250	—	—	—
Yu and Kodur (2014) [18]	CFRP	Bars	—	—	20	600	—	20	600 ^2^
Hawileh et al. (2015) [12]	CFRP	Sheets	—	—	25	300	—	25	300 ^1^
Sun et al. (2015) [20]	GFRP	Plates	93	DMA	25	125	—	—	—
Lu et al. (2016) [30]	BFRP	Plates	166.9	DMA	25	200		25	200
Ashrafi et al. (2017) [29]	CFRP	Bars	110	—	25	450	—	—	—
	GFRP	Bars	110	—	25	450	—	—	—
Jarrah et al. (2018) [10]	GFRP	Sheets	58	—	25	600	—	—	—
	CFRP	Sheets	58	—	25	600	—	—	—
Rosa et al. (2018) [3]	GFRP	Plates	104	DMA	20	180	—	—	—
Hu et al. (2018) [21]	BFRP	Plates	92	DMA	20	250	—	20	250 ^1^
Yang et al. (2019) [22]	CFRP	Plates	—	—	10	90	—	10	90
Rosa et al. (2022) [23]	GFRP	Bars	157	DMA	20	300	—	20	300 ^2^
Mazzuca et al. (2022) [24]	GFRP	Plates	94	DMA	20	250	—	20	200
Wu et al. (2023) [25]	CFRP	Bars	—	—	25	300	—	25	300 ^2^
Lu et al. (2023) [26]	GFRP	Bars	112	DSC	20	420	—	20	270 ^2^

^1^ Temperature measured at epoxy adhesive surface (FRP-concrete interface average temperatures were not provided); ^2^ Temperature measured at concrete-FRP bars.

### 2.2. Summary of Existing Thermal Properties

**Figure 3:**
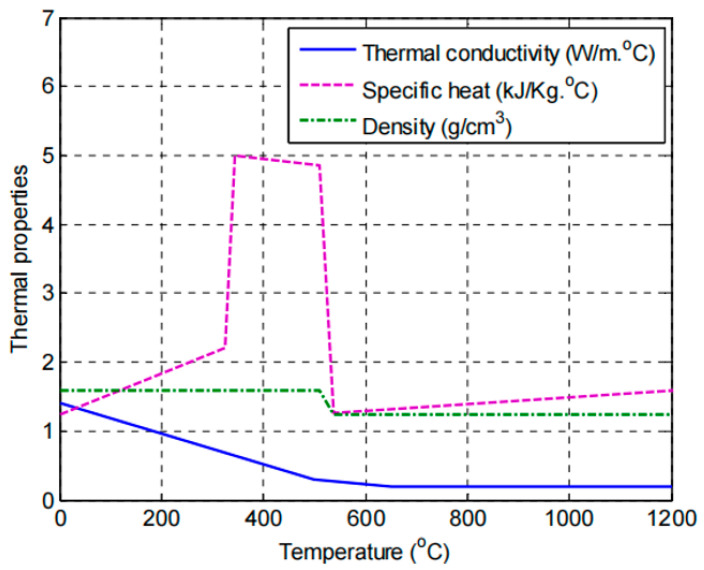
FRP Composites Thermal Properties [37].

The thermal properties of FRP composites are considered critical in their application and performance. These properties, such as density, specific heat, and thermal conductivity, are all temperature-dependent variations influencing how materials behave when subjected to elevated temperatures. FRP composites are typically composed of polymers, which are known for their relatively low thermal conductivity [36]. Compared to polymers, fibers within FRP composites have significantly higher thermal conductivity, contributing to the overall thermal behavior of FRP composites. Several factors affect FRP composites’ thermal conductivity, including fiber types, resin types, fiber orientations, and fiber volume fractions [5]. Limited experimental data on those properties are available in the literature. Griffis et al. [37] investigated the thermal response of a particular type of CFRP that had been designed for use in aerospace applications while subjecting specimens to radiant heat. Only Griffis et al.’s research [16] offers thermal properties (i.e., thermal conductivity, specific heat, and density) at extremely high temperatures (appropriately about 1200 °C), specifically above the decomposition temperature of the polymeric (epoxy) matrix, as shown in Figure 3. Therefore, understanding and characterizing FRP composite’s thermal properties at elevated temperatures is required to design FRP materials.

## 3. Bond Behavior of FRP-to-Concrete Interface at Elevated Temperatures

“Externally Bonded Reinforcement (EBR) and Near Surface Mounted (NSM)” techniques have been employed to study bond behavior between FRP-to-concrete interfaces. These techniques consist of various methods such as medium-scale measuring deformations experimental models (i.e., “single-lap shear test”, “double-lap shear test”, “direct shear test”, and “bending test”), empirical methods to characterize the best-fit bond-slip relationship through semi-analytical models, and real-scale experiment models. Besides, the fire performance of FRP-strengthened RC structures greatly influences FRP and concrete bond behavior at high temperatures based on the following interface failure modes. Adhesive degradation plays a significant role in bond strength at elevated temperatures. Epoxy-based adhesives bonding FRP to concrete rapidly degrade at temperatures between 60 and 70 °C, exceeding 100 °C, leading to severe degradation. Due to this thermal degradation, the bond between the FRP layer and the concrete leads to the eventual loss of the bond. Further, debonding is another important failure mode, which can be caused by two different mechanisms: interfacial debonding (which occurs when the shear stresses separate the FRP layer from the concrete) and adhesive failure (which occurs as a result of adhesive thermal softening or chemical changes, resulting in the adhesive losing its bonding properties). Additionally, FRP and concrete undergo differential thermal expansion at elevated temperatures, which influences interface failure modes. Temperature-induced expansion inside materials causes internal stresses at the bond interface, leading to delamination or cohesive failures (in which the concrete itself fractures or delaminates). In particular, this happens when the temperature reaches various adhesive ranges ($T_{g}$) from 50–120 °C [34,35,38]. Many experimental studies have been performed to investigate the influence of bond behavior within FRP on concrete interfaces subjected to elevated temperatures. Most of these experiments have been conducted using EBR techniques (such as [39,40,41,42,43,44,45,46,47,48,49,50,51,52,53,54,55,56,57,58,59,60]), and some limited studies have also been performed using NSM techniques (such as [42,43,46,53,58,61,62,63,64,65,66,67,68,69]). Table 2 and Table 3 summarize the collected data based on the main features of prior experimental campaigns on EBR and NSM FRP strengthening techniques.

### 3.1. Externally-Bonded Reinforcement FRP Strengthening

Blontrock [39] conducted the first-ever experiment related to “double-lap shear tests” on CFRP-strengthened laminate using epoxy adhesive with a glass transition temperature ${(T}_{g})$ quoted as 62 °C, which were performed at 40 °C, 55 °C, and 70 °C. The bonding strength obtained increased to 141%, 124%, and 82% at 40 °C, 55 °C, and 70 °C respectively. It is noteworthy that bond strength increases until the temperature approaches ${(T}_{g})$, despite this non-monotonic variation in temperature. Concrete cohesive failure occurs at room temperature. Whereas adhesive interface failure occurs when the temperature exceeds ${(T}_{g}$). As a result, bond strength significantly degrades because of the thermal degradation of the epoxy adhesive and FRP material itself under fire conditions. Klamer [41,42] investigated bond behavior between CFRP and concrete interfaces by performing “double-lap shear” and “small-scale three-point bending” tests at −20 °C to 100 °C temperature. Test results obtained in these studies indicate that ultimate load increases at the initial stage as the temperature reaches up to $T_{g}$ = 62 °C of adhesive bonding. Afterwards, bond strength decreases when the temperature increases due to adhesive softening. Additionally, failure mode happens at the debonding of aforementioned test results due to cohesive-adhesive failure within concrete (form adjacent to adhesive layer) under temperature −20 °C to 50 °C and exclusive adhesive failure occurs at elevated temperature.

Gao et al. [44] extended the mechanical loading modelling of Yuan et al. [45] and introduced closed-form expression for full-range bond performance between FRP and concrete under only the integration of mechanical and thermal conditions. Results demonstrate that the ultimate load increases with the rise in temperature, and the ultimate load decreases with the reduction in temperature. Furthermore, test results were verified with the existing test data of Klamer et al. [43] and Karter and Michael [70], and the results demonstrate close agreement. However, this model was limited to adhesive ($T_{g}$). 

Firmo et al. [46] performed “double-lap shear tests” relevant to CFRP-strengthened concrete blocks using steady-state and transient systems at 20 °C to 120 °C temperature. Investigations have also been conducted with or without mechanical anchorage influence on CFRP strip edges. Results obtained demonstrate that increasing the temperature gives rise to (i) distributions of linear axial strain at the interface, (ii) gradual bond strength depletion, (iii) increases in the effective bond length, and (iv) changes in the failure mode. For all tested temperatures, significant reductions in stiffness and maximum shear stress were observed due to bond-slip relationships at high temperatures. At 120 °C, bond strength retention was 23% under both steady-state and transient conditions.

**Figure 4:**
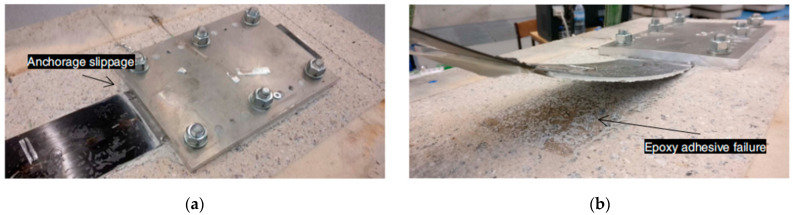
Failure modes (obtained from Correia et al. [47]). (**a**) Anchorage slippage; (**b**) Epoxy adhesive failure at elevated temperature.

**Figure 5:**
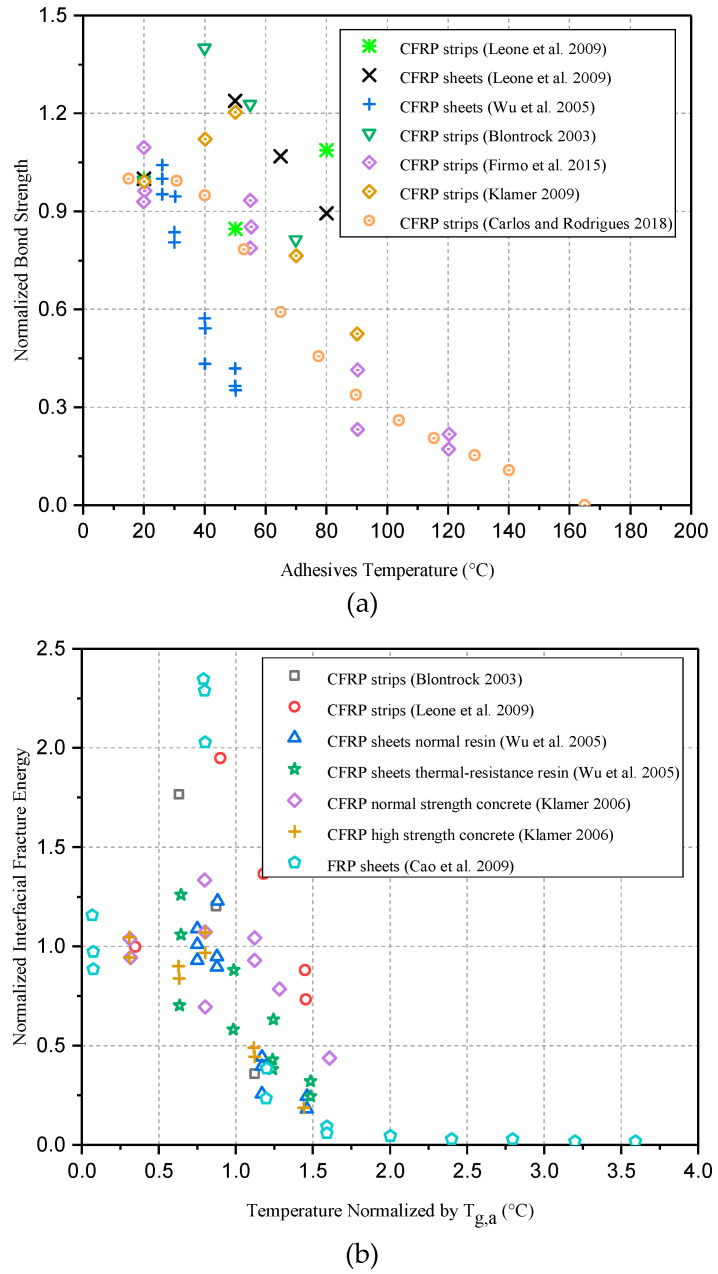
Summary of test results from the bonded joint tests at elevated temperatures. (**a**) Normalized bond strengths vs. adhesives temperatures; (**b**) Normalized interfacial fracture energy vs. temperatures (Refs. [39,40,41,42,46,48,51,54]).

Correia et al. [47] performed “direct shear tests” on (CFRP)-to-concrete blocks under 20 °C, 60 °C, and 80 °C temperatures, which were conducted by employing steady-state and transient conditions. Test results exhibit that the mechanical anchorage ultimate load capacity is reduced by about 44–59% under different normal stress levels and temperatures. Additionally, in the aforementioned test results, failure happens in regard to both the steady-state and transient configuration exhibiting laminate slippage from the anchorage at elevated temperatures because of the failure of epoxy adhesive, as shown in Figure 4. Table 2 presents a summary of existing bond joint tests on EBR interfaces between FRP-to-Concrete under elevated temperature. Figure 5 presents the comparison of previous bond tests on EBR FRP-to-Concrete interfaces (a) normalized bond strength compared with adhesives temperature (b) normalized interfacial slip distribution compared with temperature”.

**Table 2:** Summary of existing bonded joint tests on EBR FRP-to-concrete interface at elevated temperatures.

References	FRP Material	FRP Types	Bond Test	Glass Transition		Temperature [°C]	
				$T_{g}$ [°C]	Method	Min	Max
Blontrock (2003) [39]	CFRP	strips	Double lap	62	—	20	70
Wu et al. (2005) [40]	CFRP	sheets	Double lap	34–36	—	26	50
Klamer (2006, 2009) [41,42]	CFRP	strips	Double lap	62	—	−20	100
Leone et al. (2009) [48]	CFRP /GFRP	sheets /strips	Double lap	55	DSC	20	80
Burke et al. (2013) [53]	CFRP	sheets /strips	Beam tests	59 and 69	DMA	21	200
Firmo et al. (2015) [46]	CFRP	strips	Double lap	47	DMA	20	120
Ferrier and Agbossou (2017) [50]	CFRP	strips	Double lap	58 and 76	—	−40	120
KrzywoĔ (2017) [49]	CFRP	strips	Bending test	65	—	20	80
Carlos and Rodrigues (2018) [51]	CFRP	strips	Single lap	75	DMA	15	165
Correia et al. (2019) [47]	CFRP	strips	Direct shear	47	DMA	20	80
Tajmir-Riahi et al. (2019) [52]	CFRP	sheets	Single lap	—	—	25	500 ^1^
Wang et al. (2021) [56]	CFRP	sheets	Bending test	60 and 52	DSC	20	600 ^1^
Jia et al. (2021) [57]	CFRP	sheets	Single lap	68.5	—	20	120
Seo et al. (2021) [58]	CFRP	Strips	Double lap	54.5	DSC	22	411
Haris et al. (2021) [59]	CFRP	sheets	Bending test	—	—	20	350 ^1^
Khorasani et al. (2023) [55]	CFRP	sheets	Pull out	58	—	20	455 ^1^

^1^ Temperature measured at epoxy adhesive surface (FRP-concrete interface average temperatures were not provided).

### 3.2. Near Surface Mounted FRP Strengthening

Yu and Kodur [65] presented an experimental study on concrete specimens with strips and NSM CFRP bars embedding with various types of epoxy resin by performing 36 pull-out tests at a temperature ranging from 20–400 °C. The specimens have bonded with various types of epoxy adhesives such as (Tyfo T300 and Tyfo S epoxy), bars and strips, and CFRP cross-sectional shapes. Obtained test results exhibit consistent and gradual bond strength depletion by approximately 80% at 200 °C and turn out to be negligible at the temperature of 400 °C.

Burke et al. [53] investigated and compared the behavior of NSM and EBR of flexural NSM CFRP-strengthened RC beams embedding with various types of epoxy resin ($T_{g}$ = 59 °C and 69 °C obtained by DMA method) or a proprietary grout adhesive through performing 16 beam tests at a temperature ranging from 21–200 °C. Test results demonstrate that the specimen with NSM FRP-strengthened bonded with the first Epoxy failed to accomplish fire performance for several hours because of the debonding of the adhesive–concrete interface. Furthermore, this specimen continues to endure effectively for 40 min at 100 °C temperature but for less than 10 min at 200 °C temperature.

Proia et al. [67] presented a study on concrete blocks strengthened with various NSM CFRP/GFRP bars/filler systems at elevated temperatures. This study aimed to compare the test results with Palmieri [68] and Cassaert [69] on double bond shear tests to investigate the NSM bars to concrete bond interaction by employing epoxy adhesive and proprietary grout adhesive at elevated temperatures. Palmieri [68] employed the epoxy adhesive with $\left(T_{g}\right)$ of 66 °C obtained by (DSC) method. In contrast, Cassaert [69] employed the proprietary grout adhesive with ($T_{g}$) of 220 °C obtained by the (DSC) method. The results demonstrate that the reduction in bond strength of the grout was not as effective as compared to epoxy temperature. Indeed, bond strength of grout and epoxy filler is approximately 75% and 40% at 100 °C, respectively.

**Figure 6:**
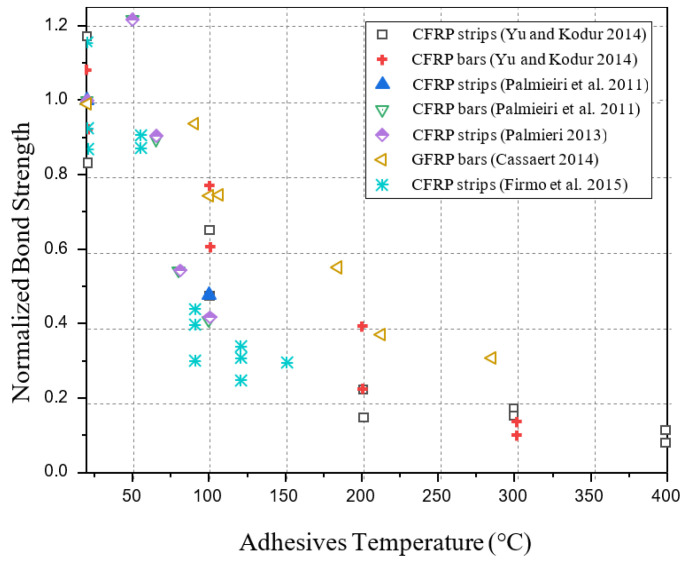
Summary of the test results of normalized bond strengths vs. adhesives temperatures (Refs. [46,65,66,68,69]).

Rosa et al. [71] investigated the bond behavior between GFRP rebars and concrete at high temperatures. Two kinds of ribbed GFRP rebars with various glass transition temperatures ($T_{g}$ = 104 °C and $T_{g}$ = 157 °C determined by DMA) were subjected to pull-out and steady-state tensile tests, from room temperature to 300 °C. Additionally, the results were then compared to fiber-wrapped-sand-coated GFRP rebars ($T_{g}$ = 98 °C) from the literature. Obtained test results indicate that at 100 °C, the maximum bond strength reduction was 34% for ribbed rebars ($T_{g}$ = 104 °C and $T_{g}$ = 157 °C), and a higher bond strength reduction of 80% for sand-coated rebars ($T_{g}$ = 98 °C) in comparison with ambient temperature strength. However, the bond strengths of all rebars were similar at temperatures above 250 °C (about 80% and 90%). Table 3 presents a summary of existing bond tests on NSM FRP-to-concrete interfaces under elevated temperatures, and Figure 6 presents the test results of normalized bond strengths versus adhesive temperatures of NSM FRP-to-concrete interfaces.

**Table 3:** Summary of bonded joint tests on NSM FRP-to-concrete interface at elevated temperatures.

References	FRP Material	FRP Types	Bond Test	Glass Transition		Temperature [°C]	
				$T_{g}$ [°C]	Method	Min	Max
Palmieiri et al. (2011) [66]	CFRP	Strips and bars	Double lap	Epoxy ($T_{g}$ = 65)	—	20	100
Burke et al. (2013) [53]	CFRP	Sheets and strips	Beam tests	Epoxy ($T_{g}$ = 59 and 69)	DMA	21	200
Palmieri (2013) [68]	CFRP	Strips	Double lap	Epoxy ($T_{g}$ = 66)	DSC	20	100
Yu and Kodur (2014) [65]	CFRP	Strips and bars	Pull-out	Epoxy ($T_{g}$ = 82 and 120)	—	20	400 ^1^
Cassaert (2014) [69]	GFRP	Bars	Double lap	Grout ($T_{g}$ = 220)	DSC	20	285
Firmo et al. (2015) [46]	CFRP	Strips	Double lap	Epoxy ($T_{g}$ = 44 and 47)	DMA	20	150
Proia et al. (2015) [67]	CFRP /GFRP	Strips and bars	Double lap	Epoxy ($T_{g}$ = 66)/Grout ($T_{g}$ = 220)	—	20	285
Lee et al. (2017) [62]	CFRP	Bars	Pull-out	Epoxy ($T_{g}$ = 50)/Grout ($T_{g}$ = 65)	DSC	−15	55
Obaidat et al. (2020) [63]	CFRP	Strips	Pull-out	—	—	23	600 ^1^
Fernandes et al. (2020) [64]	CFRP	laminate	Pull-out	Epoxy ($T_{g}$ = 73.1)	—	50	250
Seo et al. (2021) [58]	CFRP	Strips	Double lap	Epoxy ($T_{g}$ = 54.5)	DSC	22	633 ^1^
Rosa et al. (2021) [71]	GFRP	Bars	Pull-out	Epoxy ($T_{g}$ = 104 and 157)	DMA	20	300 ^2^

^1^ Temperature measured at epoxy adhesive surface (FRP-concrete interface average temperatures were not provided); ^2^ Temperature measured at concrete-FRP bars.

### 3.3. Discussion

According to the aforementioned studies, the bond behavior of the FRP-to-concrete interfaces becomes negatively impacted when exposed to elevated temperatures due to the influence of (i) the transmission of stress between FRP-to-concrete as well as (ii) the ultimate load-bearing strength of FRP composites, which are frequently encountered in civil engineering practices. Figure 5 and Figure 6 summarize conflicting findings from previous studies on the variation in bond strengths versus adhesive temperatures. Some studies show that as a result of thermal effects on both adhesive and FRP composite, bond strength within concrete-to-FRP can be significantly decreased when subjected to fire [39,40,42,48,66,68]. At temperatures around (60–70 °C), epoxy adhesives begin to degrade, whereas more significant degradation occurs above 100 °C, where the adhesive softens and loses its bonding strength [34,35,39,72]. Polyester adhesives degrade around (50–60 °C) due to their low thermal tolerance compared to other adhesives, whereas more significant degradation happens above 80 °C [34,35,72]. Although vinyl ester adhesives are more heat-resistant than polyester, their bond strength typically decreases above 80 °C, with significant degradation occurring between 100 °C and 120 °C [34,35,38]. Additionally, FRP composites also exhibit a loss of mechanical properties between 100 °C and 120 °C, causing additional degradation of the adhesive bond. Full degradation of the adhesive bond happens when temperatures exceed 150–200 °C, especially after prolonged exposure to heat, leading to a complete failure of structural integrity. Furthermore, concrete cohesive failure also causes bond strength reduction, especially at ambient temperatures, because of concrete’s inherent brittleness and microcracks, both of which are exacerbated by thermal cycling. Concrete and FRP bond strengths decrease as temperature rises due to the concrete’s internal structure deteriorating. As a result of all these factors, differential thermal expansion occurs between the adhesive, concrete, and FRP, giving rise to stress at the adhesive interface and leading to bond failure. Due to these factors, it would be beneficial to conduct further research to understand how FRP-strengthened RC structures behave under fire conditions since temperature thresholds are essential to measuring the risk of material and bond degradation. According to studies, bonds perform better at elevated temperatures when adhesives have a higher $\left(T_{g}\right)$ and by including anchorage systems. Studies have also shown that bond performance in NSM systems works better than EBR systems at elevated temperatures.

- NSM FRP-strengthened RC beams show significant retention in ultimate strength due to the degradation of their interfacial bonds after being exposed to elevated temperatures (for instance, a 53% reduction when subjected to 70 °C). Such reductions may also occur depending on the influence of FRP bar type and diameter, concrete cover, and the heat conductivity of the elements.
- Several studies above demonstrate that after temperatures exceed $\left(T_{g}\right)$, resin mechanical properties almost completely lose bond strength, leading to bond failure at elevated temperatures (for instance, 90% bond strength reduction occurs between 100 °C and 200 °C).
- Ultimate load reduction occurs after the temperature rises above $\left(T_{g}\right)$, caused by degradations in the interfacial bond during modeling. Therefore, it becomes necessary to isolate and exclude the effects of thermal stresses when interpreting bonded joint test results for such degraded interfaces at elevated temperatures.
- To enhance the ultimate strength of NSM FRP-strengthened RC members, the bond stress (shear stress) within concrete, adhesive, and FRP interfaces needs to be reduced by incorporating FRP with wider and longer measurements to increase the interface surface area between the concrete, adhesive, and FRP interfaces.

## 4. Fire Tests of FRP-Strengthened RC Members Exposed to Fire

Several studies have examined the fire performance of FRP-strengthened RC members when exposed to standard fire conditions. Nevertheless, fire performance experiments are usually defined after taking into consideration the three following performance indicators to be able to meet the specified duration: (i) insulation, (ii) load-bearing capacity, and (iii) integrity. Additionally, fire performance tests were conducted using furnace tests, which involved sustained service loads and heating scenarios. Researchers have employed various methods to determine the maximum temperature in FRP-strengthened RC members based on the test conditions and the level of accuracy desired. When it comes to detecting the temperature within the cross-sections of FRP-strengthened RC members, thermocouples are the most widely used sensors, as they offer cost-effectiveness, reliability, and real-time data. These sensors are commonly installed throughout the layers of FRP or concrete as a means of monitoring local temperatures. For surface temperature measurement, thermal cameras and infrared thermography are often used since they provide non-contact temperature measurements and capture accurate thermal maps. Fiber optic sensors, especially fiber Bragg grating (FBG) sensors, are highly accurate at measuring temperature variations within materials, including the concrete-FRP interface. Sensors like these are indispensable when monitoring temperatures in places where conventional thermocouples are not feasible. The rate of heat transfer within materials can also be measured using heat flux sensors, allowing researchers to estimate internal temperature changes and determine thermal energy distribution. Infrared sensors are commonly used in furnaces to monitor structure temperature variations, providing uniform and reliable heat exposure during testing. Table 4, Table 5 and Table 6 provide an overview of prior fire performance experiments on insulated FRP-strengthened RC members (columns, beams, and slabs).

### 4.1. Columns

In previous studies, most of the fire performance tests of FRP-strengthened RC columns have been examined when subjected to axial loads. However, relatively few fire performance tests have been performed on FRP-strengthened RC columns. Refs. [73,74,75,76,77,78,79,80,81,82] are some of the most relevant studies on the fire performance of FRP-strengthened RC columns.

Tao et al. [73] presented fire tests on FRP-strengthened circular RC column’s exposure to fire. Failure modes of these columns after fire exposure were analyzed, including (i) axial deformation of fire-damaged columns, (ii) cross-section temperatures, and (iii) fire performance of columns. Fire performance test results on FRP-strengthened circular CFST columns showed that when the temperature extended ($T_{g})$, adhesive and FRP jackets can be rubbery. It is also demonstrated that when the thickness of insulation is less than 10 mm, externally bonded FRP strength will be entirely extinct under fire conditions.

**Figure 7:**
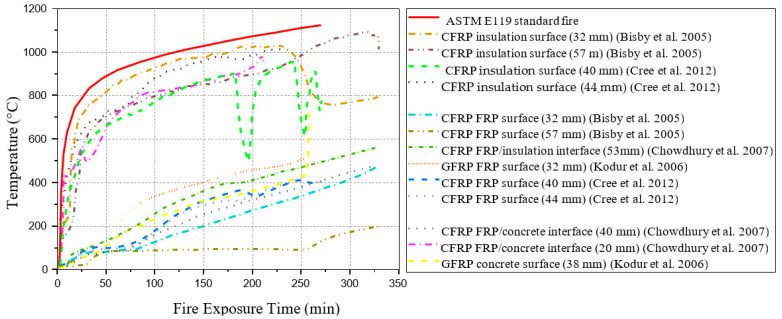
Comparisons of measured temperature responses of insulation, FRP, and FRP-to-concrete interfaces as a function of fire exposure time (Refs. [74,75,76,77]).

Bisby et al. [74] performed two full-scale fire tests on FRP-strengthened circular RC columns with fire protection insulation. The specimens were insulated with different thicknesses of “spray-applied cementitious plaster” together with the coating of intumescent epoxy at ASTM E119 standard fire. However, with the 32 mm and 57 mm thicknesses of insulations, FRP temperature was maintained below 100 °C for approximately 75 min and 180 min, respectively, under fire exposure, as shown in Figure 7. Chowdhury et al. [75] conducted a similar study on two insulated circular RC columns strengthened by CFRP with $\left(T_{g}\right)$ of 71 °C. However, the results indicate that FRP-wrapped RC columns can obtain a satisfying fire-resistance rating, even if FRP can easily be damaged under elevated temperature. Other than that, FRP-wrapped and protected RC columns can bear high temperatures for about five hours as shown in Figure 7. Additionally, in order to better understand the fire performance test results, the above-mentioned columns were compared with fire protection insulation of different thicknesses, and it has been shown that these test results can achieve fire endurance up to 90 min, as shown in Figure 7. A similar fire performance test results by Kodur et al. [76] and the results were similar to the results of Chowdhury et al. [75] and Bisby et al. [74].

Cree et al. [77] performed full-scale fire tests on CFRP-wrapped circular or square RC columns coated with a supplemental fire protection system. FRP-wrapped and protected RC columns remained unbroken for about four hours of fire performance subjected to ASTM E119 [83] and CAN/ULC-S101 [84] standard fire. A steel mesh of 3.175 mm diameter with openings of 50 mm × 50 mm was also attached to reinforce the protection system. Both specimens achieved the ultimate strengthened design capacity of 76% and 75%, respectively, according to ACI 440.2R-08 [35]. However, fire insulation failed to keep FRP temperature below its $(T_{g}$) during fire performance test. Therefore, with the 40 mm and 42 mm thicknesses of insulations, when subjected to fire tests, the FRP surface temperature was maintained below ${(T}_{g})$ onset of 60 °C for approximately 29 min and 33 min, respectively, as shown in Figure 7. Results were similar to fire performance tests observed by Kodur et al. [76], Chowdhury et al. [75], and Bisby et al. [74], even though $\left(T_{g}\right)$ of FRP composites was surpass at early stage, which is similar to test results of Benichou et al. [78].

Table 4 presents a summary of existing fire performance tests on FRP-strengthened RC columns, and Figure 7 presents the comparison between the temperature of insulation, FRP, and concrete surface/interface as the function of fire duration.

**Table 4:** Summary of fire performance tests on FRP-strengthened RC columns.

References	Column Size *h* × *b* (mm)	FRP Material	Insulation (mm)	Exposure Condition	Strength Increase [%]	Ultimate Capacity [%]
Bisby et al. (2005) [74]	400 × 400	CFRP	Vermiculite/gypsum mortar, intumescent coating (32–57)	ASTM E119	26	73
Kodur et al. (2006) [76]	406 × 406	GFRP	Vermiculite/gypsum mortar (38)	ASTM E119	10	75
Chowdhury et al. (2007) [75]	400 × 400	CFRP	—	ASTM E119 or CAN/ULC S101	63	56
Chowdhury et al. (2007) [75]	400 × 400	CFRP	Cement-based mortar (53)	ASTM E119 or CAN/ULC S101	63	56
Benichou et al. (2011) [78]	305 × 305	CFRP	Cement base mortar (40–51)	ASTM E119 or ULC S101	—	—
Cree et al. (2012) [77]	305 × 305	CFRP	Sikacretes-213F (40–44)	ASTM E119 and CAN/ULC S101	16	75
Al-Salloum et al. (2016) [81]	242 × 242	CFRP	Cement base mortar (40)	100 °C to 800 °C for 3 h	31.5	40
Sobia et al. (2022) [80]	200 × 200	CFRP	Fiber-reinforced cement composite (UHPFRCC) (25)	ASTM E119	1.50	40
Altunişik et al. (2023) [79]	20	CFRP /GFRP	DYMAT/Dymatherm (15–60)	20 °C, 200 °C, 400 °C	24	—

### 4.2. Beams

**Figure 8:**
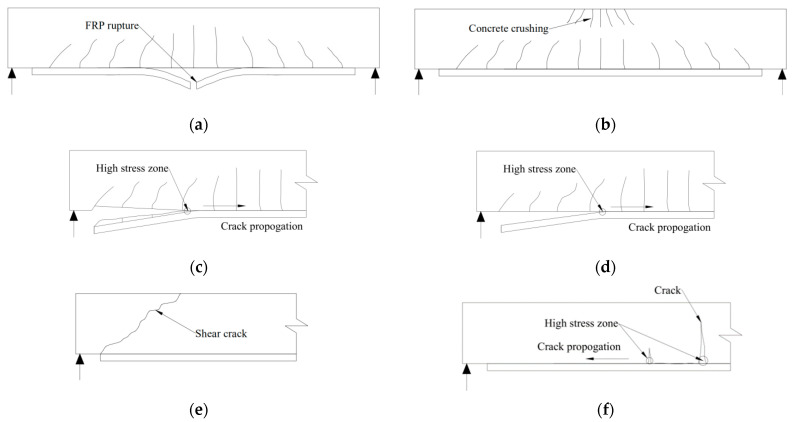
Some common failure categories of FRP-strengthened beams [97]. (**a**) Tensile rupture of FRP after steel yielding; (**b**) Concrete compression crushing; (**c**) Concrete cover delamination; (**d**) Debonding between FRP-to-concrete interface; (**e**) Shear failure; (**f**) Intermediate crack (IC) debonding.

During the past decades, numerous experimental investigations have mentioned several failure modes in RC beams strengthened through FRP (such as [39,59,85,86,87,88,89,90,91,92,93,94,95,96,97,98,99,100,101,102]). These failure modes have been broadly classified in the following categories: (i) FRP tensile rupture after steel yielding, (ii) flexural failure due to concrete compression crushing during the yielding of tension bars, (iii) concrete covering shear delamination at high-stress zones in the direction of crack propagation, (iv) debonding between FRP-to-concrete interface at the high-stress zone in the direction of crack propagation, (v) shear failure, and (vi) intermediate debonding crack produced by flexural failure (vii) axial restraint effect. Figure 8 presents a diagrammatic description of typical failure modes in prior studies.

Deuring’s study [99] conducted the first experimental study related to fire performance tests on large-scale steel/FRP-strengthened RC beams bonded with an adhesive under standard fire exposure according to the ISO 834 fire curve. Six beams of 300 mm × 400 mm and 5 m span length were studied during the experiment. One bare RC beam was considered a reference beam out of these six beams. One beam was considered a steel-strengthened RC beam. Two beams were considered CFRP-strengthened RC beams without a fire protection layer, and the other two beams were considered CFRP-strengthened RC beams by using adhesives and fire protection with various thicknesses of calcium silicate boards. Test results demonstrate that in the initial several minutes, failure occurs in the unprotected insulated CFRP-strengthened RC beams due to temperature influence, and the bond significantly degraded between concrete and FRP. However, CFRP-strengthened RC beams with fire protection layers withstand for around 120 min.

Blontrock et al. [100] performed the second experimental test on CFRP-strengthened RC beams with various insulations, such as the integration of rock wool and gypsum board, which was conducted under standard fire exposure according to the ISO 834 fire curve. This experimental study contains ten RC beams of 200 mm × 300 mm and 2.85 m span length. One un-strengthened and one strengthened RC beam were considered reference beams. Two bare RC beams were tested at service load and afterward under fire conditions. The remaining six beams were considered CFRP-strengthened RC beams that were protected with various insulation layers. Under the condition of service loads, the ultimate load-carrying capacities for un-strengthened and strengthened RC beams were around 38% and 45%, respectively. Obtained test results exhibit that all the strengthened beams expose significant bond degradation between concrete and FRP when the temperature reaches $\left(T_{g}\right)$. Furthermore, three sides U-shaped insulation applied for fire protection on RC beams exhibit better performance before the degradation of bond strength. Blontrock [39] and Kexu et al. [101] performed similar experimental studies and obtained the corresponding results.

**Figure 9:**
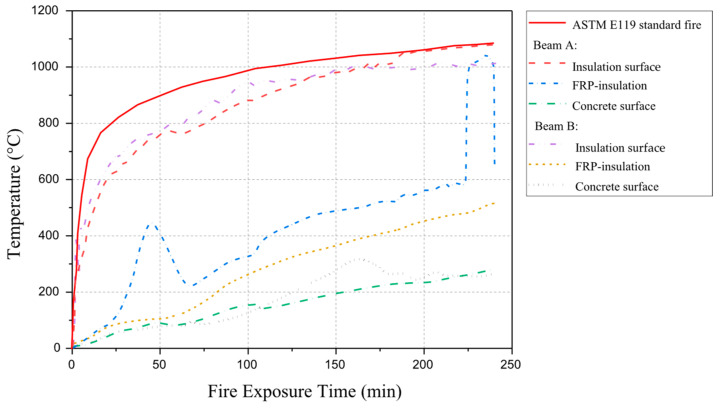
Temperature responses of beams A and B (determined by Adelzadeh [102]).

Williams et al. [85] investigated the fire performance of two large-scale RC T-beams strengthened with CFRP sheets (with an estimated 15% rise in flexural strength) and insulated using vermiculite/gypsum-based cementitious insulation (with thicknesses of 25 mm and 38 mm for Beam 1 and Beam 2, respectively). In this experiment, axial restraints were used to restrain beams at corners while they were loaded to 48% of their predicted ambient strength. Although both beams could withstand a fire for 240 min. However, beam 1’s ($T_{g}$) value approached 93 °C between 16 and 36 min, while beam 2’s exceeded between 55 and 57 min. Unfortunately, there was no clear indication of when the CFRP lost effectiveness. Adelzadeh [102] extended the research study of Williams et al. [85] and performed fire performance tests on two full-scale CFRP-strengthened T-beams with two types and dimensions of CFRP and insulation such as spray-applied cement-based mortar, which were conducted under standard fire exposure according to ASTM E119. This study aimed to investigate FRP-to-concrete interface temperature responses at mid-section span, FRP-to-insulation interface, and insulation surface. Test results demonstrate that fire performance of both beams was around 240 min. However, the bond temperature between FRP and concrete exceeded $(T_{g}$) within 30 min. However, the obtained outcomes were similar to those investigated by Williams et al. [85] except the outcomes of the performance of adhesive $(T_{g}$). It is worth noting that the axial restraint applied to the ends of the beams contributed to improved fire resistance. Figure 9 shows the distribution of temperature in both the beams as a function of fire duration.

**Figure 10:**
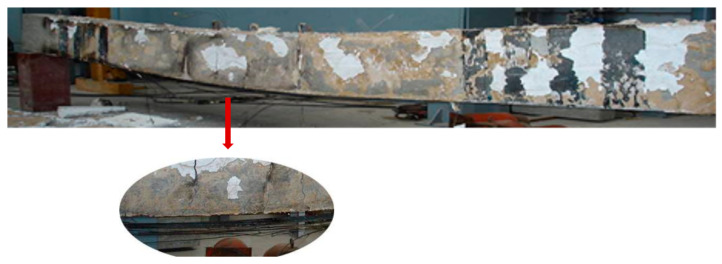
Debonding failure between FRP-to-concrete interface (obtained from Gao et al. [86]).

Gao et al. [86] conducted a series of full-scale CFRP-strengthened RC beams with externally bonded polymer adhesive ($T_{g}$ = 73 °C) and two individual types of insulation subjected to an ISO 834 fire curve. This study aimed to investigate the CFRP performance related to temperature of polymer adhesive, failure mode, end anchorage effects, deflection at mid-span, and fire performance. Test results demonstrate that fire tests for about 120 min have been accomplished by fire insulation of fire-retardant mortar coating (50 mm) or calcium silicate board claddings (40 mm). The study also indicates that by modifying the end anchorage, there will be an improvement in the performance of CFRP-strengthened RC beams. Figure 10 shows the debonding within the FRP and concrete after removing the insulation layers.

Yu and Kodur [103] tested four full-scale RC CFRP (strips)-strengthened T-beams using (NSM) procedures and were subjected to ASTM E119 fire test using a four-point bending test. During the test phase, the beams were subjected to different loading levels (50% or 65% of their predicted ambient strength), a 25 mm fire insulation system made of vermiculite gypsum plaster, and axial restraint influence. As a result, CFRP strips debonded from the beam center early in the fire exposure; however, due to the cooler anchorages, tension was maintained. Consequently, the strengthening system remained structurally effective for 210 min of fire exposure.

**Figure 11:**
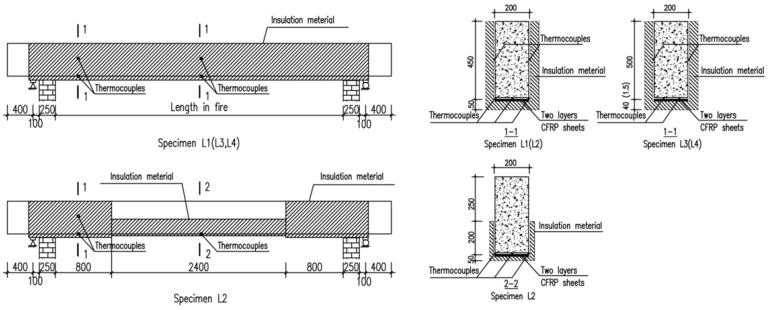
Diagram of thermocouples and fire insulation schemes (obtained from Dong et al. [87]).

**Figure 12:**
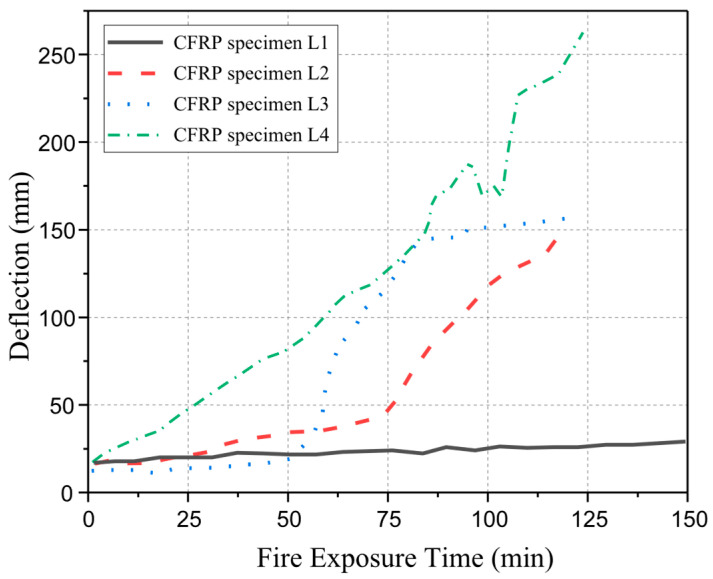
Measured deflection at midspan responses as a function of fire duration [87].

Dong et al. [87] presented a series of full-scale CFRP-strengthened RC beams protected with various insulation layers exposed to the ISO 834 fire curve. During the experimental studies, beams 1 and 2 have cross-sections of 200 mm × 450 mm and span lengths equal to 4.7 m, and beams 3 and 4 have cross-sections of 200 mm × 500 mm and 5.2 m span lengths. The beams were fire-insulated with an ultra-thin coating (1.5 mm), calcium silicate board (40 mm) on the entire length, and thick mortar coating (50 mm) at the anchorage zone. Thermocouple and fire insulation specifications are shown in Figure 11. This study aimed to examine the thermal and structural responses of insulated CFRP-strengthened RC beams. Test results demonstrated that the fire performance of insulated CFRP-strengthened RC beams was around 120 min, as shown in Figure 12. CFRP-strengthened RC beams provide better protection using a U-shaped protection system. Table 5 presents a summary of existing fire performance tests on FRP-strengthened RC beams.

**Table 5:** Summary of fire tests on FRP-strengthened RC beams.

References	Beam Size L × *h* × *b* (mm)	FRP Material	Insulation (mm)	Exposure Condition	Strength Increase [%]	Ultimate Capacity [%]
Deuring (1994) [99]	5000 × 400 × 300	Carbon	Calcium silicate boards (40–60)	ISO 834	—	55
Blontrock et al. (2000) [100]	2850 × 300 × 200	Carbon	Gypsum boards/rock wool (25–60)	ISO 834	—	38
Kexu et al. (2007) [101]	5500 × 450 × 200	Glass	Cement-based mortar (20–50)	ISO 834	—	—
Williams et al. (2008) [85]	3900 × 400 × 300	Carbon	Vermiculite/gypsum mortar (25–38)	ASTM E119	15	48
Gao et al. (2010) [86]	5500 × 450 × 200	Carbon	Calcium silicate board, fire retardant coatings (40–50)	ISO 834	—	—
Ahmed and Kodur (2011) [88]	3960 × 406 × 254	Carbon	Vermiculite/gypsum mortar (25)	ASTM E119	50	50
Firma et al. (2011) [89]	2100 × 120 × 100	Carbon	Calcium silicate boards, vermiculite/perlite mortar (25–40)	ISO 834	94	47
Adelzadeh (2013) [102]	3900 × 400 × 300	Carbon	Cement base mortar (40)	ASTM E119	63	56
Firmo and Correia (2015) [91]	1500 × 120 × 100	Carbon	Calcium silicate boards (25–75)	ISO 834	74	36
Dong et al. (2016) [87]	4700 × 200 × 450	Carbon	Ultra-thin coating, calcium silicate board, cement base mortar (1.5–40–50)	ISO 834	—	—
Zhang et al. (2018) [90]	5300 × 400 × 250	Carbon/Boron	Primer layer (10)	ISO 834	—	—
Carlos et al. (2018) [92]	3400 × 300 × 150	Carbon	Vermiculite-perlite mortar, clay aggregates, cement-based mortars (20–35–50)	ISO 834	60	48
Bhatt et al. (2021) [94]	3960 × 406 × 254	Carbon	V-Wrap FPS (19–32)	ASTM E119	33	55
Haris et al. (2021) [59]	2885 × 400 × 200	Carbon	Cement-based mortar (20)	ISO 834	24	30
Dong et al. (2023) [93]	6000 × 450 × 250	Carbon	Cement-based mortar, intumescent fire-retardant coating (20)	ISO 834	09	29

### 4.3. Slabs

Williams et al. [104] conducted fire tests on four intermediate-scale FRP-strengthened RC slabs containing different CFRP sheets externally bonded with epoxy adhesives ($T_{g}$ = 82 °C) and three individual types of insulation exposed to ASTM E119 fire conditions. The slabs were fire-protected with fire insulation such as VG insulation (fire-resistant plaster applied using spray) with thicknesses of 19 mm and 38 mm employed on the surface of CFRP wraps and afterward trowel-coated the Tyfow EI coating (intumescent epoxy surface-hardening coating) of 0.25 mm thickness on VG insulation surface. Test results obtained in this study indicate that the four-hour fire performance test has accomplished with the appropriate design and four insulation schemes with a thickness of 38 mm. Furthermore, the required time to reached $(T_{g}$) was 42–104 min, which depends on the applied fire-protected fire insulation. More recently, Stratford et al. [105] presented similar results to Williams et al. [104], in which the slab under fire exposure was only subjected to self-weight, signifying that specimens do not prevent any realistic evaluation of FRP structural effectiveness.

López et al. [106] conducted six fire performance tests with intermediate-scales to study CFRP-strengthened RC slabs fire performance (length × wide × deep equal to 2.10 m × 0.10 m × 0.12 m, respectively) externally bonded with epoxy adhesives ($T_{g}$ = 55 °C), protected with or without passive fire protection systems. The slabs held strengthening in flexural, with an increase in strength of approximately 94%. The above-mentioned design strategy presented an exceptional temperature reduction of the strengthening system. Furthermore, the recommended design strategy guarantees the following: (i) CFRP strengthening laminate temperature kept maintained below a definite limit such as 500 °C and (ii) CFRP-to-concrete interface temperature remains beneath the epoxy resin ($T_{g}$ = 55 °C) along the anchorage zone.

Bakhtiyari et al. [107] examined five intermediate-scale insulated RC slabs with CFRP strips strengthening, bonded with epoxy resin, and exposed to the BS EN 1363-1:2012 [108] temperature–time curve. The five slab specimens were subjected to fire performance tests, which included two insulated strengthened specimens, two uninsulated strengthened specimens, and one unstrengthened specimen. Due to dimensional limitations, one-quarter scaling factors were scaled for the test specimens in order to perform fire performance tests in a one-meter cube furnace. The slab specimens are fire-protected with fire insulation with gypsum-vermiculite mortar (Vermifire-G sprayed coated) with thicknesses of 15 and 25 mm. Test results of this study revealed that CFRP strips can enhance the flexural strength of slabs. Slabs’ load capacity rises to 228–247%. Failure deformation and ductility of slabs exhibit improvement owing to the presence of CFRP strips. The fire performance of CFRP-strengthened concrete slabs improves from 11 to 74 min because of the applied fire protection systems. However, the epoxy resin used during the bonding of the CFRP-to-concrete interface has failed to keep its integrity when the temperature becomes close to or above $(T_{g}$). This has led to the detachment of continuous carbon fibers. Table 6 presents a summary of existing fire tests on FRP-strengthened RC columns.

**Table 6:** Summary of fire tests on FRP-strengthened RC slabs.

References	Slab Size L × *h* × *b* (mm)	Technique	FRP Material	Insulation (mm)	Exposure Condition	Strength Increase [%]
Williams et al. (2006) [104]	950 × 150	EBR	CFRP	Vermiculite/gypsum mortar, Cement base mortar (15–40)	ASTM E119	—
Stratford et al. (2009) [105]	150	NSM and EBR	CFRP	Intumescent coating, Gypsum boards (24)	ASTM E119	—
Bakhtiyari et al. (2017) [107]	1460 × 50 × 980	EBR	CFRP	Vermiculite/gypsum mortar (15–25)	BS EN 1363-1:2012	228–247
López et al. (2018) [106]	2100 × 120 × 250	EBR	CFRP	Vermiculite/perlite mortar, Calcium silicate boards (25–40)	ISO 834	94
Rosa et al. (2020) [109]	1500 × 110 × 250	NSM	GFRP	Ceramic wool	ISO 834	60
Bhatt et al. (2021) [94]	3960 × 152 × 400	EBR	CFRP	V-Wrap FPS (19–25)	ASTM E119	40
Azevedo et al. (2022) [110]	1500 × 110 × 250	NSM and EBR	CFRP	Calcium silicate boards (25–50)	ISO 834	81 and 18

### 4.4. Discussion and Future Recommendations

Several prior research results were reviewed and discussed in the section mentioned above. These include (1) glass transition temperature $\left(T_{g}\right)$ and adhesion properties; (2) FRP strengthening RC members subjected to fire; (3) shear strength of laminate interface via concrete and load-bearing strength; and (4) the effect of fire protection systems. The performance of insulation plays a crucial role in preventing structural damage caused by fire, particularly in FRP-strengthened RC members. Fire-resistant insulation effectiveness depends greatly on its thermal conductivity. Mineral wool, calcium silicate, or intumescent coating materials have low thermal conductivity and provide better heat retention. Meanwhile, gypsum-based or vermiculite boards offer substantial protection in high-temperature environments of up to 1000 °C. However, it is critical to note that environmental factors, including humidity, moisture exposure, and mechanical stress, can adversely affect insulation thermal properties with time. The presence of moisture can reduce the effectiveness of insulation, and certain materials, such as fiberglass, become less fire-resistant. Additionally, long-term exposure to UV radiation leads to thermal cycling, and chemical degradation further reduces its durability. Therefore, to ensure long-term fire protection and structural integrity, it is essential to carefully select and apply fire-resistant insulation materials suited to the structure’s environmental conditions. This review aims to enhance the fire performance and strength capacity of FRP-strengthened RC members based on the following findings, with future research recommendations also included:

- All the columns insulated with fire insulation successfully maintained their full-service load-bearing capacity for more than four hours, although the glass transition temperature $\left(T_{g}\right)$ of FRP being surpassed early in the test.
- The above research studies demonstrated that FRP laminates significantly degrade tensile and bonding properties when exposed to fire. Due to this degradation, the stiffness and strength of FRP-strengthened RC beams were significantly reduced. Moreover, these studies indicate that FRP-strengthened RC beams can achieve the required fire performance criteria by using appropriate fire insulation systems. Yet, it remains unclear how to design insulation systems appropriate for FRP-strengthened RC beams based on the number of fire tests that have been conducted.
- With the application of 20-mm thick fire-retardant coating (i.e., SJ-2 layer), CFRP shear-strengthened RC beams can effectively delay the cross-sectional temperature responses of the test specimens, leading to the enhancement in the fire resistance period.
- According to this review of literature, it has been observed that there has been a lack of studies on FRP-strengthened RC members subjected to cyclic and impact loading at elevated temperatures to determine their dynamic behavior. The majority of experiments have been performed under static loading conditions.
- Using real fire for experimental studies has rarely been reported since most studies have relied on electric furnaces. During real fires, structural elements will behave differently than what has been observed during artificial fire experiments.
- To conduct experimental fire testing outdoors on FRP-strengthened RC members to examine a real case scenario considering wind and air environmental conditions.
- Fire performance tests are required for RC structural members externally strengthened with CFRP strips of zigzag shape at the bottom and side anchorages.
- To conduct experimental fire testing on insulated FRP-strengthened RC members using mineral fibers.
- To conduct experimental fire testing on the fire performance of RC beams strengthened in shear with CFRP at elevated temperatures.
- Fire performance investigations are required for CFRP-strengthened RC structural beams reinforced by a combined mechanically anchored 3NSM system.
- To conduct experimental fire testing on U-shaped steel anchorage plates applied in the mid-section of CFRP-strengthened RC beams to determine the second proposed design approach.
- An experimental fire test is required to be conducted on a pre-stressed CFRP strengthening device.
- Fire performance tests are required to mechanically anchor RC beams strengthened with CFRP laminates by combining π-anchor and FRP anchor devices.

## 5. Numerical Modelling of FRP-Strengthened RC Members Exposed to Fire

Due to the significant difficulties involved, only a few attempts at numerical modeling have been made in the literature to determine the fire performance of FRP-strengthened RC members under standard fire tests. Nevertheless, numerical modeling on the fire performance of FRP-strengthened RC members exposed to fire based on the following five performance indicators are taken into account: (i) fire exposure time vs. temperature, (ii) fire exposure time vs. axial deformation, (iii) mid-height deflection vs. axial load, (vi) bond degradation influences, and (v) insulation thickness impact. The following sections provide an overview of the numerical modeling of currently available FRP-strengthened RC members (columns, beams, and slabs).

### 5.1. Columns

In previous studies, the Finite Element (FE) approach has been effectively employed to predict heat transfer analysis of bare RC columns, as demonstrated in references [111,112,113,114]. Meanwhile, the FE approach has been applied to analyze FRP-strengthened RC structures, as shown in the research efforts of [74,82,115,116,117,118].

Bisby et al. [115] presented a numerical procedure for evaluating the fire performance of insulated FRP-wrapped RC circular columns, as well as un-insulated FRP-wrapped RC circular columns under fire conditions. The objective of this modeling was to perform an analysis of (i) the axial strength of the model under strain-equilibrium conditions subjected to standard fire exposure and (ii) heat transfer analysis using the finite-difference method. Bisby et al. [115] determine the ultimate moment capacity of a column at its mid-height, assuming that the distance ratio between the center of the column and the point of application of the load to the column’s width is equal to 1.0. Nevertheless, the model exhibited strong agreement with the findings of Bisby et al. [74], demonstrating conclusions such as the relationship between time and axial load capacity, as well as the correlation between mid-height deflection and axial load during fire exposure. Chowdhury et al. [116] developed a similar numerical simulation and compared the outcomes of FE modeling that were adopted for thermal analysis proposed by Bisby et al. [115] as well as the Column Deflection Curve (CDC) approach for modeling the structural behavior of 2-D fiber-element analysis proposed by Chen and Atsuta [117]. The purpose of the study was to predict the impact of FRP-wraps on the column’s corners, as well as the non-linear mechanical and thermal behaviors of the columns, specifically the second-order moments. In addition, this simulation not only predicts the progression of axial load measurements in FRP-wrapped RC rectangular columns but also predicts the efficiency of FRP durability.

**Figure 13:**
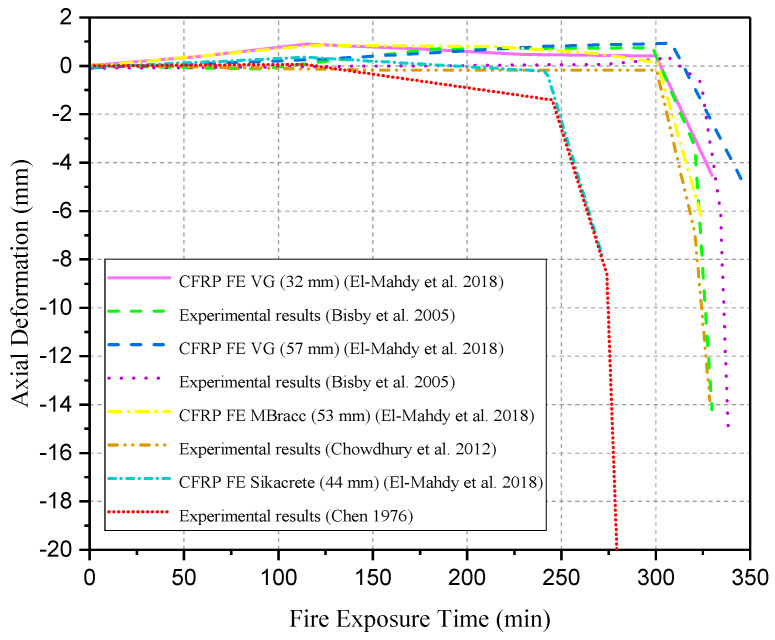
Axial deformations of columns as a function of fire duration (Refs. [115,116,117,118]).

El-Mahdy et al. [118] conducted a simulation of a model to determine the behavior of Insulated FRP-strengthened RC columns under the combined effects of fire and service load. The models were coated with a fire-insulating coating. The purpose of the study was to predict the effect of FRP wraps on the corners of columns, as well as the non-linear mechanical and thermal performance of the columns, specifically the second-order moments. The numerical simulation of these numerous FRP-confined and insulated RC columns shows good agreement with experimental results in terms of axial deformation and temperature distribution. In addition, the predicted axial deformation of the columns was compared to experimental data from published experimental works under fire and service load conditions for a specific period of time, as illustrated in Figure 13.

### 5.2. Beams

Several numerical studies were conducted to analyze the mechanical and thermal response of FRP-strengthened RC beams when subjected to fire. Thermal analysis in these numerical analyses was often simulated using either the finite element (FE) approach or the finite difference approach [119,120,121,122,123,124,125,126,127,128,129]. Alternatively, the mechanical behavior of bare RC beams has also been analyzed employing either the FE approach or standard sectional analysis [119,121,122,125,126,128,130,131]. Hawileh et al. [132] analyzed the structural and temperature distribution responses of externally strengthened RC T-section beams insulated with FRP through the nonlinear FE approach by utilizing the results data gathered from fire tests conducted by Williams et al. [85]. Moreover, the validated and developed FE simulation was expanded to investigate the impact of different sizes and types of FRP materials, as well as the ratio of reinforcement bars (rebars). Kodur and Ahmed [133] performed a thermal analysis to investigate the structural performance of externally FRP-strengthened beams with insulation during fire conditions. Some of the numerical studies stated above assumed that rebars and externally bonded FRP laminate were completely bonded with concrete. However, it is common to observe debonding failure between concrete and FRP in FRP-strengthened RC beams [98,134,135].

Dai et al. [136] developed a nonlinear local bond-slip model to investigate the behavior of FRP laminates (EB) on concrete at high temperatures. In this model, temperature-induced thermal stress and bond degradation are integrated into a two-parameter bond-slip model based on ambient temperature. Two parameters were determined from shear test data for FRP-concrete-bonded joints under elevated temperatures, such as interfacial fracture energy ($G_{f}$) and interfacial brittleness index (*B*). During lower temperatures, ($G_{f}$) remained relatively constant until it started to drop as the adhesive reached its ($T_{g}$) level. A similar trend was observed in the interfacial brittleness index (*B*). The proposed model of temperature-dependent bond slip effectively simulates the test data and has the potential to be used in theoretical analysis of the fire performance of FRP-strengthened RC members despite test data variability.

Gao et al. [121] developed a 3D (FE) model for predicting the fire performance of insulated FRP-strengthened RC beams, a key concern for building construction. During the FE approach, FRP, concrete, and steel material behaviors, along with their bond–slip interactions at FRP-to-concrete and steel-to-concrete interfaces, are modelled carefully. Based on a comparison between the model’s predictions and actual experiment data, the model proved to be accurate, demonstrating that previous numerical approaches that assumed bonding between FRP and concrete miscalculated deflections and fire resistance. As a result of this study, a performance-based fire safety design approach can be used for parametric studies to simplify the design of FRP-strengthened RC members in fire scenarios.

**Figure 14:**
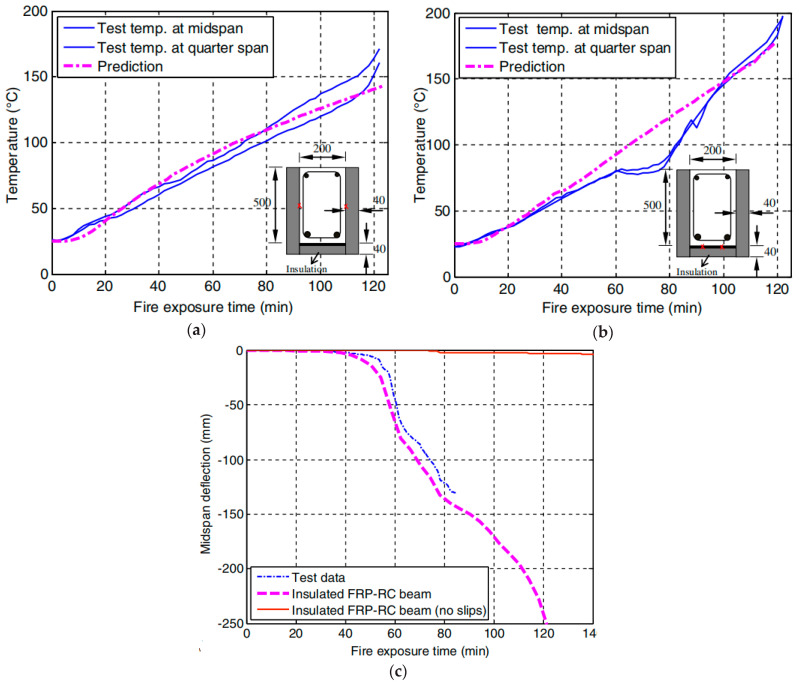
FE model predictions of insulated CFRP-strengthened RC beams of Gao et al. [86]. (**a**) mid-height temperature; (**b**) bottom surface temperature; (**c**) midspan deflection.

Dai et al. [137] developed a three-dimensional finite element simulation to analyze the behavior of insulated FRP-strengthened RC beams under fire conditions while considering the degradation of the bond between FRP reinforcement and internal steel due to temperature by incorporating data from fire tests of [85,86,100] to accurately model the structural and thermal behavior. Figure 14 illustrates the fire performance of insulated CFRP-strengthened RC beams, as simulated by Gao et al. [86].

### 5.3. Slabs

Several numerical studies have been undertaken to approach the fire performance validates that are of concern for FRP-strengthened concrete slabs [104,105,106,138,139]. The aforementioned experiments considered characteristics such as the slab thickness, the configuration of insulation, fire scenarios, methods of strengthening, and load levels. These studies suggest that in order to enhance the fire performance of reinforced slabs, it is important to provide fire insulation. The tests demonstrate that the failure of strengthened slabs is caused by bond degradation between concrete and FRP due to the influence of temperature. Additionally, the temperature curve of insulated FRP-strengthened RC slabs put through a full-scale fire test has been performed by several authors (i.e., [106,138,139]) via comprehensive numerical modeling. The purpose of these simulations is to predict the ideal thickness of insulation for strengthened slabs, specifically at the steel rebars or FRP-concrete interface, under specific temperature criteria.

Bisby and Kodur [140] performed numerical modeling to accurately predict the fire performance of RC slabs subjected to fire conditions. During the numerical analysis, the outcomes show significant distinctions in steel RC and FRP-strengthened slab performance. Moreover, the analysis has been executed to investigate the influence of different types of FRP bars, overall slab thickness, and slab cover. Furthermore, this study proposed a simple numerical methodology to analyze the key differences between steel RC slabs via FRP-strengthened slabs.

Hawileh et al. [141] performed numerical simulations to examine the structural performance of one-way GFRP RC slabs at elevated temperatures. During this analysis, strength and serviceability specifications regarding slab design were conceded from the ACI-440.1R [142]. Based on numerical analysis, an approach has been demonstrated to enhance the fire performance of the slab by including two layers of FRP reinforcement.

**Figure 15:**
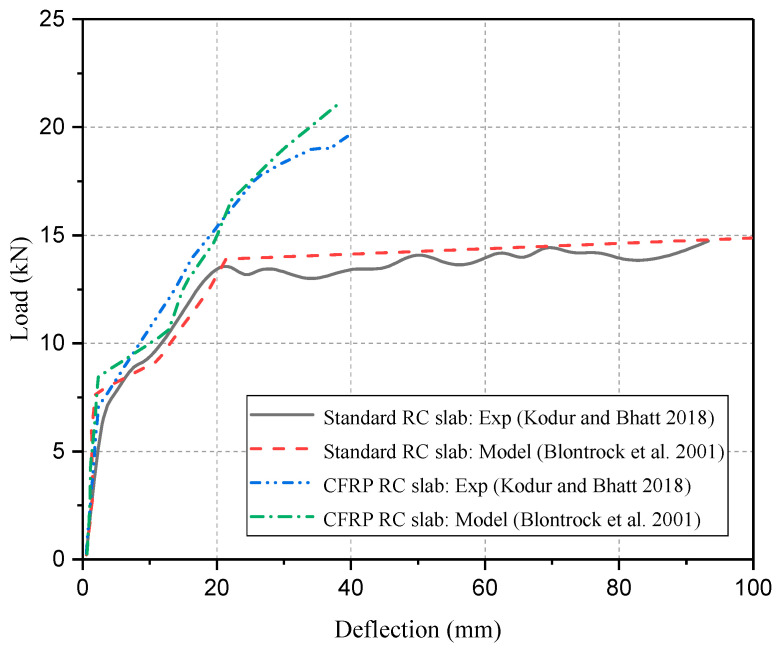
Comparisons of measured and predicted load-deflection responses at ambient temperature (Refs. [138,143]).

Kodur and Bhatt [143] conducted numerical modeling to evaluate the performance of FRP-strengthened RC slabs subjected to structural loading and fire exposure. The numerical modeling included temperature-dependent properties of FRP, concrete, steel, and fire insulation, as well as temperature-influenced bond degradation between FRP and concrete interface. Moreover, the simulation employs a macroscopic finite element-based approach to compute the thermo-mechanical response from the linear-elastic stage to a failure state. The outcome of this numerical modeling demonstrates that RC slabs without insulation have poor fire performance. Furthermore, the comparison between the above results and the experimental outcomes of Blontrock et al. [138] has been illustrated in Figure 15.

### 5.4. Discussion and Future Recommendations

According to the above literature review, FRP-strengthened RC members are well-suited to continuous integration into civil engineering applications. A wide range of construction applications can be accommodated with FRP composites due to their superior properties. Unfortunately, accurately simulating the behavior and response of FRP-strengthened RC members appears to be complicated, which may prevent designers from fully capitalizing on the potential of these composite components. In FE modelling, one of the main challenges that occur when simulating is the debonding process between FRP laminates and concrete surfaces. Consequently, we faced such a challenge due to limited data available for a coefficient of friction as well as the appropriate scale of contact stiffness values. Further, when FRP-strengthened RC members are exposed to high temperatures, they encounter additional simulation challenges. This is mainly due to the fact that there is insufficient data available on the thermal and mechanical behavior of FRP composites; hence, certain assumptions have to be made. Moreover, the simulation of FRP splay anchors presents additional issues because of their complex geometry and the lack of comprehensive data related to bonding between anchor-to-concrete as well as anchor-to-FRP interfaces. According to Dai et al. [137], prior numerical studies have consistently neglected the impact of steel-concrete bond degradation under fire conditions. Existing modeling approaches have several limitations that affect their accuracy and reliability. One of the most significant limitations concerns determining FRP materials’ thermal expansion coefficient as a function of temperature. FRP materials usually embed fibers in a resin, which leads to complex and temperature-dependent expansion properties. Numerical modeling often fails to incorporate the actual changing effect of the FRP thermal expansion coefficient at elevated temperatures. FRP materials have an anisotropic nature, which means their properties behave differently in different directions (i.e., longitudinal versus transverse), which makes modeling more complicated. These directional properties vary non-uniformly at elevated temperatures, and prior numerical simulations rarely consider their impact on the overall structural performance of FRP-strengthened RC members. Further, bonding behavior at FRP-concrete interfaces is another significant limitation for the structural performance of FRP-strengthened RC members. Although the interface’s bond behavior changes with temperature, many numerical models, unfortunately, simplify the bond-slip relationship or assume constant bond strength, making further investigation necessary. According to our previous discussion, adhesive properties at the interface degrade as temperatures rise above the ${(T}_{g}$) level, resulting in the FRP system debonding or delaminating. These complex, temperature-dependent bond mechanisms lack adequate representation in many models, leading to inaccurate failure mode predictions at high temperatures. Dai et al. [136] propose a nonlinear local bond-slip analysis for FRP laminates externally bonded to concrete that can be used in the theoretical prediction of fire performance of RC structures strengthened with FRP at elevated temperatures. Gao et al. [121] and Dai et al. [137] propose a 3D FE model for predicting interfacial bond-slip behavior at concrete-to-steel interfaces not previously considered. Furthermore, the decoupled analysis approach is another significant limitation that should be considered. The decoupled analysis approach is most commonly used, according to which thermal analysis is performed first, followed by a mechanical simulation based on the resulting temperature field as input. Although this method simplifies calculations, the results are not realistic. During actual fires, fire penetrates deeper into the material once cracks form in the structure under fire conditions, affecting the inside temperature distribution. This dynamic interaction process among thermal and mechanical behavior has been ignored during decoupled analyses, which consider a static temperature field. Therefore, the model may not accurately predict temperature distribution variation under fire, especially in already cracked areas. As a result, these models may not accurately predict realistic structural responses. As an example, FRP debonding from the concrete surface often occurs as a result of localized high temperatures, which decoupled models cannot simulate. Further research is needed in the following areas:

- Heat transfer analysis on FRP-strengthened RC elements at varying temperature conditions, including both cold and hot temperature conditions within the cross-section of the members.
- Finite element analysis (FEA) will investigate the creep-rupture behavior of FRP-strengthened RC members under sustained loads. A viscoelastic model can incorporate concrete creep under sustained loading, whereas a viscoelasticity or viscoplasticity model can be used to model FRP degradation over time (i.e., polymer matrix creep).
- Time-dependent analysis to investigate the long-term deflection response of flexural strengthened RC elements with various kinds of FRP components under sustained loads. As part of this analysis, time-varying material properties (i.e., concrete creep, shrinkage, and stress relaxation in FRP) can be incorporated to predict deflections under sustained loads.
- Numerical analysis to evaluate the impact of lightweight concrete on the shear and flexural behavior of FRP-strengthened RC members.
- Simulating high-strength concrete to study its influences on shear and flexural behavior of FRP-strengthened RC members.
- Simulating RC beams and slabs externally strengthened with FRP splay anchors to investigate the static, cyclic, and thermal loading conditions.
- Heat transfer analysis to predict fire performance of FRP shear-strengthened RC beam at elevated temperature.

## 6. Design Methods for Temperature Predictions

### 6.1. Existing Fire Resistance Design Methods

During the past decade, several experimental and numerical studies have been performed to examine the behavior of FRP-strengthened RC members at various temperatures. Regarding these analyses, several design methods have also been proposed for the structural development of FRP-strengthened members (i.e., [35,144,145,146] However, the design guidelines, as mentioned earlier, take into account some or all of the following requirements: (1) specify the parameters for determining the maximum load capacity and load combinations to be considered in the design for fire performance; (2) provide guidance for the overall fire performance design process; (3) evaluate the performance of the FRP-strengthening system under fire protection conditions; (4) instructions for responding to fire safety. To improve the performance of bare RC members, some authorities have only admitted the implementation of FRP after verifying the full strength of FRP under fire for a prolonged duration. However, fire insulation layers are required to protect FRP-strengthened RC members and achieve fire resistance requirements. Therefore, implementing fire insulation in the design guidance lines is highly recommended. For different types of buildings, the design of fire protection systems in design guidelines must include restrictions on using FRP materials to prevent extremely rapid flame spread and the emission of smoke. During the production process, the majority of commercial FRP materials are coated or formulated to meet the requirements of nearly all building regulations. Designing for structural integrity is particularly tough during a fire scenario. Therefore, conducting fire tests is considered a good strategy due to its safety perspective. However, conducting such fire tests appears difficult due to their lack of economic feasibility. To develop such a procedure, modern concepts of exhaustive fire design tests have to be followed. Although a performance-based approach is the most suitable for fire performance designs, these methods are complex and time-consuming. Therefore, simple design-oriented approaches are needed to predict the temperatures of bare RC members and FRP-strengthened RC members insulated with fire insulation layers.

#### 6.1.1. Prescriptive Design Guidelines

Prescriptive methods for fire performance design are derived from empirical data obtained through fire testing. Several design codes have provided preliminary guidance for the fire performance of structural members (i.e., ACI Committee 440 [142], ACI 440.2R-08 [35], Concrete Society [147], CSA [148]). Prescriptive methods mainly provide precise guidelines for the thickness of concrete cover and approximate dimensions of RC elements to satisfy the criteria of fire performance design. For the majority of traditional buildings, this method relies directly on the principles of mechanics, failure modes, the behavior of reinforcement in concrete under high temperatures, and design assumptions. Nevertheless, this approach may not be economically viable and is not applicable to elements that have been strengthened or fortified with FRP. Therefore, the Prescriptive approach is unsuitable for FRP material due to a lack of sufficient study.

#### 6.1.2. Performance-Based Fire-Resistance Design

Performance-based design methods have been given priority in recent years, bringing more consideration to fire safety than recurrent and modern prescriptive-based methods. In doing so, the performance-based design method has achieved appropriate fire safety resolution and cost-effectiveness. Performance-based design encompasses numerous characteristics. Among these crucial characteristics, the fire-resistant design offers the most significant notion, which has undoubtedly provided remarkable results in structural design. This method employs two approaches to analyze and evaluate the fire behavior of structural elements. One approach involves the use of mathematical models that simulate the conditions. Another approach involves formulating design formulas derived from mathematical models. These design methods can be utilized to incorporate building codes.

In North America, ACI 216 [149] has been acknowledged as the most effective method for conducting analysis, as recommended by ACI 440.2R [35]. However, such standards are rare and can only be deemed appropriate in particular circumstances. While CAN/CSA S806-12 [145] provides design guidelines for FRP reinforcement in concrete structures, it is based on the principles of limit state design and closely aligned with the National Building Code of Canada (NBCC) [150]. However, failures of these approaches occur because of the combustibility of FRP, which contributes to the fuel source and consequently increases the fire intensity of the concrete surface. Considering the aforementioned concepts, it is necessary to incorporate insulation into fire prevention schemes for performance-based procedures. Fib Bulletin 14 [144] illustrates such an approach’s fundamental effectiveness by incorporating insulation into fire prevention schemes.

### 6.2. Proposed Simple Design Methods for Temperature Predictions

During fire exposure, it is requisite to have accurate temperature predictions for FRP-strengthened RC members to accomplish a cost-effective and reliable fire insulation scheme. In general, after creating the design of the fire insulation scheme, we can conduct temperature analysis by using the procedure of finite element (FE) or finite-difference (FD) employed by [85,115,116,133,137]. However, it will be difficult for researchers to accomplish the complex design task because of the restricted time period for the computations of the simulation. Therefore, to ensure a reliable fire-resistant design for these insulated components, it is crucial to accurately predict the distribution of a time-varying temperature to have a simple design-oriented method.

#### 6.2.1. Bare RC Members

Several studies have been performed on “simple design-oriented methods” for accurately predicting temperatures in RC members directed to 1-D heat transfer analysis (Harmathy [151] and Kodur et al. [152]) or 2-D heat transfer analysis (Wickstrom’s [153], Desai [131], Abbasi [154], Abbasi and Hogg [155], Kodur et al. [152]). According to ISO 834, RC beams were made using standard-weight concrete and exposed to a standard fire test. However, Wickstrom’s study [153] predicted that the temperature trend would rise ∆*T*(*x*, *y*) at the provided points (*x*, *y*). The equations shown below demonstrate the correlation between temperature increases and their corresponding relationships:

(1) $$\Delta T(x,y)=[n_{w}(n_{x}+n_{y}-2n_{x}n_{y})+n_{x}n_{y}]\Delta \theta_{f}$$

here, ∆$\theta_{f}$ expresses the increase in temperature as per the ISO-834 fire curve. The variable $n_{w}$ represents in Equation (1) the ratio between the temperature rise of the fire and the beam surface temperature rise, depending on the duration of the fire. The equation of $n_{w}$ is defined as:

(2) $$n_{w}=1-0.0616t^{-0.88}$$

Equation (2) represents the time period *t* (in hours) in terms of fire exposure. Whereas the variables $n_{y}$ (or $n_{x}$) are functions of fire duration and provide a ratio between the reference value (*α_c_* = 417 × $10^{9}\;m^{2}$) and thermal diffusivity of the RC beam presented as (*α*) is given below:

(3) $$n_{x}=0.18\ln(\frac{\alpha }{\alpha_{c}}\times \frac{t}{x^{2}})-0.81$$

where the variable *x* (in m) represents the distance between the fire-exposed surface along with the consideration point in the width direction of the beam. By substituting the variable *x* with *y* in the equation mentioned above, the values of $n_{y}$ can be calculated in the vertical direction of the beam. The above equation is valid only on the condition that it satisfies Equation (4):

(4) $$x(or)y\geq 2h-3.6(0.0015t{)}^{0.5}$$

where *h* (in m) represents the height of the RC beam in the under-consideration direction.

Furthermore, Eamon and Jensen [156] adopted Wickstrom’s [153] approach to acquire 500 °C isotherm and the necessary temperature data from the literature to ensure the fire endurance of prestressed RC beams. Furthermore, Desai [131] proposed that the isotherms of RC beams more often align parallel to the exposed surface of RC beams and additionally assumed simple equations. In addition, Desai [131] also recommended three main key factors: fire exposure duration, beam breadth, and the ratio between the height and weight of RC beams. Additionally, Abbasi and Hogg [155] have conducted research on the prediction of temperature for FRP reinforcement in beams exposed to fire. They presented a theoretical formula based on the ISO 834 standard fire curve. The study found that the temperature of the reinforcements was influenced by fire duration and concrete cover thickness. In their recent work, Kodur et al. [152] made modifications to Wickstrom’s [153] equation and introduced simple equations to predict temperatures within the cross-section of RC members based on the ASTM E119 [83] and ISO 834 [157] standards. These modified equations fulfilled the requirement of previous equations. The temperature at fire exposure time in RC members can be determined using the following Equations (5)–(7):

One-dimension heat transfer equation for slabs:

(5) $$T=c_{1}\cdot n_{x}-(at^{n})$$

Two-dimension heat transfer equation for RC columns and beams:

(6) $$T=c_{1}\cdot [-1.481\cdot (n_{x}\cdot n_{y})+0.985\cdot (n_{x}\cdot n_{y})+0.017]\cdot (at^{n})$$

where the values of the coefficients $c_{1}$ and $c_{2}$ depend on the concrete strength and type of aggregates. The variable $n_{x}$ represents the functions of fire duration in the direction from the consideration point to the exposed surface. The term ${at}^{n}$ represents the value of the proposed standard fire curve. The expression is defined as:

(7) $$n_{x}=0.155\ln(\frac{t}{x^{1.5}})-0.384\sqrt{x}-0.371$$

where *x* (in m) represents the distance between the fire-exposed surface and the point being considered in the width direction of the member, and *t* (in h) represents the fire duration. Meanwhile, a similar equation was also introduced to determine $n_{y}$ in the vertical direction of the member. The author suggested that the temperature rises after member parts are subjected to impacts at −20 °C.

Gao et al. [158] developed a simple, design-oriented approach for predicting temperatures in bare RC beams exposed to standard fire conditions, capable of being implemented on uninsulated FRP-strengthened RC beams. In this approach, columns, slabs, and walls are classified as 1D or 2D heat transfer regions according to heat transfer boundaries and sectional characteristics. Based on Gao et al. [158], the temperature tends to rise Δ*T* at a specific depth *d* (mm) in bare RC member subjected to standard fire from one-side (for instance, the bottom side width is more extensive than 600 mm) can be demonstrated as below:

(8) $$\Delta T=\theta_{d,120}k_{t}k_{b}$$

where $k_{b}$ considers the impact values of consideration point in the width direction of RC members higher than 600 mm, $k_{t}$ consider the impact values of fire duration *t* (in min), and $\theta_{d,120}$ show reference temperature tends to rise at t14. The formulas of these expressions are shown below:

(9) kb=exp⁡[b0+b1b/200+b2ln⁡(b200)]

(10) $$k_{t}=\frac{t_{1}t_{2}+t_{3}t^{t_{4}}}{t_{2}+t^{t_{4}}}$$

(11) $$\theta_{d,120}=a_{0}\cdot \exp(a_{1}d)+a_{2}$$

In the above equations, the values of the parameters $a_{0},b_{0,}a_{1},b_{1},a_{2},b_{2}$ and $t_{i(1,2,3,4)}$ are actual functions for concrete depth provided by Gao et al. [158] exposed to a standard fire from two sides. Two-dimension temperature response can be determined through Equation (12), modified by Gao et al. [158], as shown below:

(12) $$\Delta T=\{[\ln(\frac{\theta_{x}}{\theta_{y}}+1)]\cdot \theta_{y}\cdot m(y)\cdot n(\frac{y}{x}){\}k}_{t}k_{b}$$

In this equation, *x* represents the larger distance, while *y* represents the smaller distance between the exact location. These distances are being considered in relation to the surface that has been exposed to fire. The values of $\theta_{x}$ and $\theta_{y}$ can be found using Equation (12) by substituting *d* with *x* and *y*. In accordance with Gao et al. [158], the logarithm expression of Equation (12) can be used to describe the interaction within the two perpendicular directions with respect to heat transfer. The equations *m*(*y*) and $n(\frac{y}{x})$ represent the interaction effects of these two directions on heat transfer and are defined as follows:

(13) $$m(y)=0.759+4.37\times 10^{-3}y-1.71\times 10^{-5}y^{2}$$

(14) $$n\left(\frac{y}{x}\right)=1.26-1.32\left(\frac{y}{x}\right)+0.881{\left(\frac{y}{x}\right)}^{2}$$

#### 6.2.2. Insulated FRP-Strengthened RC Members

Several studies have been carried out on simple formula evolution to have accurate predictions of temperatures for insulated steel members under fire exposure. Comprehensive descriptions and evaluations of such a variety of formulas can be obtained somewhere else (Refs. [159,160,161]). However, the aforementioned formulas are mostly achieved by determining a simplified one-dimension problem of heat transfer and employing the “lumped heat capacity method” (Refs. [160,161,162]). Since steel has high thermal conductivity, the “lumped heat capacity method” stands suitable for steel; however, the method mentioned earlier is not suitable for fire-insulated RC members mainly because of two ACI 440.2R [35] principles: (i) the temperature variation between the insulated member surface and the fire is essential and should not be ignored; (ii) in the concrete section, the temperature gradient is usually essential. Admittedly, steel does not have a functional heat absorption capability compared to concrete since concrete can absorb and store heat energy effortlessly due to its low thermal conductivity. Furthermore, the “lumped heat capacity method” considers immediate and consistent heat transfer throughout the structure, overlooking the reality that temperature variations occur in large or thick materials such as concrete, resulting in incorrect predictions of temperature throughout the member. Due to concrete’s low thermal conductivity, heat transfers through it slowly and unevenly, resulting in a lag between surface and internal temperatures, which lumped models cannot capture. Furthermore, it ignores material heterogeneity, such as concrete and steel reinforcement’s different thermal expansion rates, resulting in internal stresses and structural damage during fire conditions.

Based on the above limitations, Gao et al. [163] proposed a relatively “simple design-oriented method” to have accurate and simple temperature prediction for insulated FRP-strengthened RC members exposed to standard fire. Gao et al. [163] extended Gao et al. [158] method and proposed a method using a Microsoft Excel spreadsheet calculation for insulated FRP-strengthened RC members in which the fire insulation layer is replaced with an equivalent concrete layer. As a result, an insulated RC member’s temperature analysis is equivalent to that of a bare RC member, resulting in a wider cross-section formed by an equivalent concrete layer. The key challenge is to determine the equivalent concrete layer’s thickness, which must be related to the fire insulation layer’s thickness and thermal properties. The results clearly show that variations in thermal conductivity affect temperature response more than changes in volumetric heat capacity. Furthermore, the FE approach for determining the temperature of insulated FRP-strengthened RC members was modelled and verified with existing test data providing accurate results. The Equations (15) and (16) below demonstrate the acquisition of $k_{\lambda }$ and $k_{\rho c}$ through the process of least-squares optimization.

(15) $$k_{\lambda }=e_{1}\cdot (\frac{\lambda_{a}}{0.1}{)}^{e_{2}}$$

(16) $$k_{\rho c}=f_{1}+f_{2}\cdot (\frac{\rho_{a}c_{a}}{750})$$

where the terms $e_{1},e_{2},f_{1},\;and\;f_{2}$ present as functions of insulation layer thicknesses, and these expressions can be obtained using the following equations.

(17a) $$e_{1}=-9.93\times 10^{-1}-3.55\times 10^{-3}d_{i}+1.05\times 10^{-4}d_{i}^{2}-2.55\times 10^{-7}d_{i}^{3}$$

(17b) $$e_{2}=-9.23\times 10^{-1}+3.08\times 10^{-2}d_{i}-8.45\times 10^{-4}d_{i}^{2}+6.53\times 10^{-6}d_{i}^{3}$$

(17c) $$f_{1}=9.75\times 10^{-1}+4.57\times 10^{-4}d_{i}-6.84\times 10^{-5}d_{i}^{2}$$

(17d) $$f_{2}=1.64\times 10^{-2}+6.84\times 10^{-4}d_{i}+4.59\times 10^{-5}d_{i}^{2}$$

The thicknesses of equivalent concrete layers have been intentionally determined using the above equations. In this method, the total fire exposure time is broken down into small time steps, where temperature responses are determined step-by-step depending on equivalent concrete layer thickness and insulation thermal properties calculated from a Microsoft Excel spreadsheet. Nevertheless, this approach has significant limitations. Firstly, the method can only be applied to standard fire exposures and not to design fires, such as those specified by EN 1991-1-2 [164], involving a cooling stage. Secondly, assuming that concrete contains only 1.5% moisture by weight, the model ignores moisture migration; thus, the temperatures may be slightly overestimated. Finally, there is a possibility that the method may not be accurate for RC beams exceeding 200 mm in width since heat transfer may occur from more than two surfaces, making temperature distribution challenging.

Recently, Kodur et al. [165] presented that substituting the equivalent distance *y*′ and *z*′ (concrete width or depth) into temperature equations of RC members can achieve a temperature rise within the insulated RC members, as given in the equations below:

(18) $$z^{\prime }=z_{c}+z_{ec}=z_{c}+z_{i}{\sqrt{t}}^{\beta }\sqrt{\frac{k_{c}}{(\rho c{)}_{c}}\frac{(\rho c{)}_{i}}{k_{i}}}$$

(19) $$y^{\prime }=y_{c}+y_{ec}=y_{c}+y_{i}\sqrt[{a}]{t^{\beta }}\sqrt{\frac{k_{c}}{(\rho c{)}_{c}}\frac{(\rho c{)}_{i}}{k_{i}}}$$

Heat transfer equation in one dimension:

(20) $$T_{c}=c_{1}\cdot n_{x}\cdot (at^{n})$$

(21) $$\eta_{z}=0.155\ln\frac{t}{(z^{\prime }{)}^{1.5}}-0.348\sqrt{z^{\prime }}-0.371$$

Heat transfer equations for RC beams and columns in two dimensions:

(22) $$T=c_{1}\cdot [-1.481\cdot (n_{x}\cdot n_{y})+0.985\cdot (n_{x}\cdot n_{y})+0.017]\cdot (at^{n})$$

In the above equations, *z*′ expresses the distances between the surface of fire to the point in the concrete section, while *t* (in hours) expresses the fire duration. Whereas $\eta_{z}$ and $\eta_{y}$ indicate the heat transfer coefficients originating from *z*′ and *y*′. However, the determination of $\eta_{y}$ has been resolved using the same course of action as $\eta_{z}$ in Equation (21). Equations (18)–(22) are regarded as equivalent to those defined in the equations of Wickstrom’s [153]. They are used to clarify the expression of temperatures in the insulating RC beam. However, *α* and *β* are only the unknowns that are needed to be resolute in Equations (18) and (19). Moreover, the value of *α* and *β* can be acquired through the database generated by utilizing finite element analysis (FEA).

### 6.3. Results and Discussion

According to studies conducted by Gao et al. [163], an accurate yet simple, approximate method has been presented to predict the thermal behavior within bare or insulated FRP-strengthened RC elements at standard fire conditions. FRP-strengthened RC elements typically require protection by utilizing external fire insulation in order to improve their resistance to fire. By taking into consideration thermal properties and fire insulation thickness, it is possible to convert the layer of fire insulation applied to RC elements to an equivalent-thickness concrete layer. Due to this transformation, we can more accurately determine the overall fire performance characteristics of the structure [166]. Thus, after conducting a comprehensive parametric assessment with regard to one-dimensional heat transfer into both bare and insulated RC elements based on a validated finite element method, the correlation between the thickness of fire insulation and the equivalent concrete layer has been established. Additionally, a design example illustrates the effectiveness of the simplified approach to predicting temperatures within the cross-section of insulated RC beams at standard fire conditions. The applicability and accuracy of the developed approach have been validated with a large dataset of temperature results from finite element analysis and fire tests, which partially validates the validity of this approach. As a result of the proposed equations, temperatures within the cross-section of insulated RC elements subjected to standard fire conditions are likely to be predicted within a margin of ±10%. A simple approach like this one has great potential for practical application due to its simplicity.

## 7. Conclusions and Future Research

The present study provides a State-of-the-Art review of the fire performance of FRP materials (including polymer matrix) and FRP-strengthened RC members. The review has revealed that over the past few decades, numerous research efforts have been executed in terms of experimental studies such as mechanical, thermal, and bond properties of FRP at elevated temperatures and fire tests of FRP-strengthened RC members, numerical simulation, analytical methods (i.e., proposed a simple design method for temperature predictions), and existing design methods for fire performance (i.e., prescriptive design guidelines and performance-based fire-resistance design approach). A detailed analysis of the fire performance of FRP-strengthened RC members has led to the following conclusions and future research recommendations:

1. Although fiber plays an important role among FRP composites to sustain the applied load, the mechanical properties of the polymer matrix (i.e., $\left(T_{g}\right)$, ultimate strain, tensile strength, elasticity modulus strength, and bond strength between the matrix and fiber) are also essential in influencing the mechanical behavior of FRP composites. In addition, some other significant factors that affect the bond and tensile properties of FRP strengthening systems and materials include thermal cycling, the effect of curing conditions, absorption of moisture, aging and creep, and sustained loading.
2. Based on the experimental studies performed on the mechanical properties of FRP materials used for strengthening at elevated temperatures, it can be concluded that bond strength reduction is considered an important factor in not obtaining the required response of FRP-strengthened RC members at elevated temperatures. However, the degradation in bond strength is mainly due to FRP having lower fire performance itself. Therefore, several researchers have concluded that by applying a certain level of insulation, FRP-strengthened RC members can achieve the required fire endurance rating.
3. Experimental studies conducted at elevated temperatures play a significant role in the bond behavior of FRP-to-concrete interfaces. The increase in the ultimate load is observed for the bonded joint subjected to a warm temperature close to $T_{g}$, due to the influence of initial thermal stresses occurring at the FRP-to-concrete interface. Eventually, the degradation has been observed for the ultimate loads of the bonded joints tested at elevated temperatures owing to the degradations of the bonding adhesive, which can be accurately explained by a temperature-dependent bond-slip model.
4. Fire tests and numerical simulations on insulated FRP-strengthened RC members have indicated that the test specimens can achieve satisfactory fire endurance ratings once appropriate fire insulation layers are utilized.
5. The current design guidelines for FRP-strengthened RC members usually provide some prescriptive provisions for fire endurance design, which cannot be easily implemented in practical strengthening applications. Therefore, a simple design method has been reviewed and proposed for more rational fire endurance design approaches for FRP-strengthened RC members under fire conditions.
6. The above review reveals that almost no research work in the literature has been conducted on the fire performance of FRP shear-strengthened RC beams, and therefore, it is an urgent need to carry out some fire tests and related numerical modeling of FRP shear-strengthened RC beams under fire conditions.
7. Further research is necessary to produce adhesives with higher thermal resistance and stronger bonding at high temperatures to prevent adhesive degradation and ensure stronger, better bond strength at FRP-to-concrete interfaces under fire conditions.
8. Considering the limitations of current FRP materials, to improve the fire performance of FRP-strengthened RC structures, further research on hybrid FRP systems that combine carbon, glass, and aramid fibers may offer better fire performance.
9. For the fire safety of FRP-strengthened RC members during high temperatures, further investigation is required on innovative fire protection strategies, including intumescent coatings, insulating layers designed to resist fire, and passive fire protection systems.

## Data Availability

Data are contained within the article.
